# Adenosine mediates functional and metabolic suppression of peripheral and tumor-infiltrating CD8^+^ T cells

**DOI:** 10.1186/s40425-019-0719-5

**Published:** 2019-10-10

**Authors:** Beatris Mastelic-Gavillet, Blanca Navarro Rodrigo, Laure Décombaz, Haiping Wang, Giuseppe Ercolano, Rita Ahmed, Leyder Elena Lozano, Angela Ianaro, Laurent Derré, Massimo Valerio, Thomas Tawadros, Patrice Jichlinski, Tu Nguyen-Ngoc, Daniel E. Speiser, Grégory Verdeil, Nicolas Gestermann, Olivier Dormond, Lana Kandalaft, George Coukos, Camilla Jandus, Christine Ménétrier-Caux, Christophe Caux, Ping-Chih Ho, Pedro Romero, Alexandre Harari, Selena Vigano

**Affiliations:** 1 Department of Oncology, Ludwig Institute for Cancer Research Lausanne, Lausanne University Hospital and University of Lausanne, Lausanne, Switzerland; 2 Department of Oncology, University of Lausanne, Lausanne, Switzerland; 3 Department of Pharmacy, University of Naples Federico II, Naples, Italy; 4 Department of Urology, Urology Research Unit, CHUV, Lausanne, Switzerland; 5 Department of Visceral Surgery, CHUV, Lausanne, Switzerland; 6 Department of Immunology Virology and Inflammation, Univ Lyon, Université Claude Bernard Lyon 1, 69008 Lyon, France; 7 INSERM 1052, CNRS 5286, Centre Léon Bérard, Cancer Research Center of Lyon, Lyon, France

**Keywords:** Adenosine, CD8 T cells, Metabolism, mTOR, TILs

## Abstract

**Background:**

Several mechanisms are present in the tumor microenvironment (TME) to impair cytotoxic T cell responses potentially able to control tumor growth. Among these, the accumulation of adenosine (Ado) contributes to tumor progression and represents a promising immunotherapeutic target. Ado has been shown to impair T cell effector function, but the role and mechanisms employed by Ado/Ado receptors (AdoRs) in modulating human peripheral and tumor-infiltrating lymphocyte (TIL) function are still puzzling.

**Methods:**

CD8^+^ T cell cytokine production following stimulation was quantified by intracellular staining and flow cytometry. The cytotoxic capacity of tumor infiltrating lymphocytes (TILs) was quantified by the chromium release assay following co-culture with autologous or anti-CD3-loaded tumor cell lines. The CD8^+^ T cell metabolic fitness was evaluated by the seahorse assay and by the quantification of 2-NBDG uptake and CD71/CD98 upregulation upon stimulation. The expression of AdoRs was assessed by RNA flow cytometry, a recently developed technology that we validated by semiquantitative RT-PCR (qRT-PCR), while the impact on T cell function was evaluated by the use of selective antagonists and agonists. The influence of Ado/AdoR on the PKA and mTOR pathways was evaluated by phosphoflow staining of p-CREB and p-S6, respectively, and validated by western blot.

**Results:**

Here, we demonstrate that Ado signaling through the A2A receptor (A2AR) in human peripheral CD8^+^ T cells and TILs is responsible for the higher sensitivity to Ado-mediated suppression of T central memory cells. We confirmed that Ado is able to impair peripheral and tumor-expanded T cell effector functions, and we show for the first time its impact on metabolic fitness. The Ado-mediated immunosuppressive effects are mediated by increased PKA activation that results in impairment of the mTORC1 pathway.

**Conclusions:**

Our findings unveil A2AR/PKA/mTORC1 as the main Ado signaling pathway impairing the immune competence of peripheral T cells and TILs. Thus, p-CREB and p-S6 may represent useful pharmacodynamic and efficacy biomarkers of immunotherapies targeting Ado. The effect of Ado on T cell metabolic fitness reinforces the importance of the adenosinergic pathway as a target for next-generation immunotherapy.

**Electronic supplementary material:**

The online version of this article (10.1186/s40425-019-0719-5) contains supplementary material, which is available to authorized users.

## Background

Recently, it was discovered that activated T cells undergo a pronounced mTOR-mediated metabolic switch [1–3], and failure to reprogram metabolic activities leads to a hyporesponsive state, including exhaustion and anergy [4, 5]. In the tumor microenvironment (TME), multiple mechanisms such as overexpression of immune checkpoint molecules [6] and loss of nutrients [7, 8] were recently shown to impair T cell metabolic fitness, thus impairing immune control of tumor growth [9, 10]. However, it remains largely unexplored whether and how immunosuppressive molecules, such as adenosine (Ado), impair T cell functions by subverting their metabolic activity.

Extracellular Ado is produced by the sequential dephosphorylation of ATP catalyzed by two cell surface ectonucleotidases, CD39 and CD73. In the TME, the ATP released by cell death, the increased expression of CD39 and CD73 and the decreased expression of the adenosine deaminase (ADA)/CD26 complex lead to the accumulation of Ado [11, 12]. The persistent high concentration of Ado may become detrimental, generating an immunosuppressive microenvironment [13–18]. Mechanistically, Ado mediates multiple effects by binding the G-protein-coupled receptors A1, A2A, A2B and A3 [19]. Signaling of A2A and A2B adenosine receptors (A2AR, A2BR) through Gs proteins increases cyclic AMP levels and protein kinase A (PKA) activity [19–21]. Studies with selective agonists/antagonists have revealed the key role of A2AR in the suppression of T cell functions [15–17, 20]. However, the relative sensitivity of T cell subsets to Ado and the relevant mediators downstream of Ado receptors (AdoRs) have not yet been determined.

Here, we demonstrate that Ado immunosuppressive effects on human CD8^+^ T cells are primarily exerted on T central memory cells (T_CM_), likely due to their higher levels of A2AR expression. Selective blockade of A2AR, but not A2BR, restored CD8^+^ T cell functionality. Triggering A2AR increased PKA activation (i.e.*,* CREB phosphorylation), resulting in impaired TCR signaling, the mTORC1 (but not mTORC2) pathway (i.e.*,* S6 phosphorylation), cytokine production and metabolic fitness, both in the context of T cell polyclonal stimulation and of tumor cell recognition and killing by autologous tumor infiltrating lymphocytes (TILs).

Our findings unveil the A2AR/PKA/mTORC1 pathway as the main axis for the Ado-mediated impairment of T cell function and metabolic fitness. In line with other studies evaluating the Ado pathway as a relevant target for immunotherapy [13, 14, 16, 21], we corroborate that blockade of A2AR has great potential for next-generation immunotherapy, and we propose p-CREB and p-S6 as potential biomarkers of efficacy for validation in future clinical studies.

## Methods

### Subjects and specimen preparation

Human blood samples from healthy donors were collected at the local Blood Transfusion Center Lausanne, Switzerland, under IRB approval (Ethics Committee, University Hospital of Lausanne-CHUV). Written informed consent was obtained from all healthy subjects and patients, in accordance with the Declaration of Helsinki. Fresh anticoagulated blood diluted at a 1:2 ratio in PBS was layered on lymphoprep (ratio of diluted blood:lymphoprep 1.5:1). Mononuclear cells were isolated by density gradient centrifugation (1800 rpm, 20 min centrifugation without break, room temperature), washed twice and immediately cryopreserved in 90% fetal calf serum (FCS) and 10% DMSO.

Informed consent from the cancer patients was obtained based on the procedures approved by the same IRB as mentioned above. Clinical characteristics are described in Additional file 2: Table S2.

Freshly resected tumors not needed for histopathologic diagnosis were transferred in transport media (RPMI + 2% penicillin-streptomycin) in sterile containers at 4 °C. Tumors were then cut into 1–2 mm^2^ pieces and used freshly or cryopreserved in 90% human serum+ 10% DMSO.

### Antibodies and reagents

Anti-CCR7 (CD197) Alexa Fluor 488 (clone G043H7), anti-CCR7 (CD197) PE/Cy7 (clone G043H7), anti-CD107a (LAMP-1) Brilliant Violet 510 (clone H4A3), anti-CD16 Alexa Fluor 700 (clone 3G8), anti-CD19 Brilliant Violet 650 (clone HIB19), anti-CD3 Brilliant Violet 605 (clone UCHT1), anti-CD3 APC/Fire750 (clone SK7), anti-CD4 Brilliant Violet 421(clone RPA-T4), anti-CD4 PE/Dazzle 594 (clone RPA-T4), anti-CD45RA Alexa Fluor 700 (clone Hl100), anti-CD56 PE (clone NCAM), anti-CD71 PE/Cy7 (clone CY1G4), anti-CD73 PE/Dazzle 594 (clone AD2), anti-CD8 PE/Cy7 (clone RPA-T8), anti-CD8 Brilliant Violet 650 (clone RPA-T8), CD8 FITC (clone SK1); anti-IL2 PE (clone MQ1-17H12), anti-PD1 (CD279) Brilliant Violet 421 (clone EH12.2H7), and anti-TNF-α PE/Cy7 (clone Mab11) were purchased from BioLegend. Anti-CD39 Brilliant Violet 711 (clone TU66), anti-CD4 BUV496 (clone SK3), anti-CD45RA Brilliant Violet 510 (clone Hl100), anti-CD8 Pacific Blue (clone RPA-T8), anti-CD98 PE (clone UM7F8), and anti-IFN-γ APC (clone B27) were purchased from Becton Dickinson. The Anti-phospho-CREB^Ser133^ Alexa Fluor 647 (clone 87G3), unconjugated anti-phospho-S6^Ser235/236^, and unconjugated anti-phospho-Akt^Ser473^ (clone 193H12) were purchased from Cell Signaling Technology. The secondary antibody goat anti-rabbit IgG H&L Alexa Fluor 488 was purchased from Abcam. Stable adenosine (Ado), A2AR agonist CGS 21680, A2AR inhibitor ZM 241385, A2BR inhibitor PSB 1115, PKA inhibitor KT 5720, rapamycin and AKT_1/2_ inhibitor MK 2206 were purchased from Sigma-Aldrich. Anti-CD3 used for the redirected cytotoxicity assay was generated by a hybridoma in house.

### Virus-specific peptides

A peptide pool of CMV-EBV-Flu-specific peptides optimal for CD8^+^ T cells has been purchased from JPT Peptide Technologies. The following HLA-A2-restricted single peptides have been synthetized by the peptide facility at the Ludwig Cancer Institute of Lausanne: CMV-NLVPMVATV, EBV-GLCTLVAML, and Flu-GILGFVFTL.

### Cell cultures

#### Cell lines

The EBV-transformed B-cell line (generated in house), human leukemic lines: THP1, (catalog number (cn): TIB-202, ATCC) and HL60 (cn: CCL-240, ATCC), prostate cancer lines: DU145 (cn: HTB-81, ATCC) and PC3 (cn: CRL-1435, ATCC), LNCaP (cn: CRL-1740, ATCC), breast cancer cell line MCF-7 (cn: HTB-22, ATCC) and the mastocytoma cell line P815 (cn: TIB-64, ATCC) were maintained in tissue culture flasks in RPMI supplemented with 10% FCS, amino acids and HEPES. All cell lines were periodically tested for mycoplasma contamination and confirmed negative by PCR with mycoplasma-specific primers (5′-ACTCCTACGGGAGGCAGCAGTA-3′ and 5′-TGCACCATCTGTCACTCTGTTAACCTC-3′).

#### Human peripheral blood mononuclear cells (PBMCs)

PBMCs were cultured in RPMI supplemented with 5% penicillin-streptomycin, 25 mM HEPES, and 8% heat-inactivated FBS. When mentioned, assays were performed in glucose-free RPMI supplemented with 5% penicillin-streptomycin.

#### Tumor-infiltrating lymphocytes (TILs)

For TILs expansion, tumor tissues were dissected into fragments of approximately 2 mm^3^. Each fragment was plated individually in a single well of a 24-well plate and stimulated with 6000 IU/ml rhIL-2 for 3 weeks. A rapid expansion protocol (REP) was performed by stimulating TILs with PHA 1 μg/ml, 3000 IU/ml rhIL2 (Proleukin, Roche) and feeders. TIL culture media was RPMI supplemented with 5% penicillin-streptomycin (Gibco), 25 mM HEPES, 1% L-glutamine (Gibco), 1% nonessential amino acids (Gibco), 1% Na pyruvate (Gibco), 0.1% 2β-mercaptoethanol (Gibco), and 8% heat-inactivated, pooled human serum.

#### Melanoma cell lines

Autologous tumor cell lines were established from three melanoma samples. Tissue was mechanically dissociated, with a clamp and scalpel, and enzymatically dissected using collagenase type I (Sigma-Aldrich) and deoxiribonuclease I (Roche) for 45–60 min at 37 °C. Tumor cell suspensions were cultured in RPMI, 10% fetal bovine serum (Gibco), and 1% penicillin-streptomycin (Gibco), dispensed into 10-cm petri dishes, and transferred to T-25 and T-75 flasks as cells expanded.

### TIL/autologous tumor cell line cocultures

When described, cells were preincubated with the A2AR antagonist ZM 241385 (30 μM) or the A2BR antagonist PSB 1115 (100 μM) for 1 h and 30 min and then with nothing or Ado (30 μM) for 2 h. After preincubation, 200,000 TILs/well were dispensed into a 96-well plate containing, or not, the same number of autologous melanoma tumor cells. Intracellular staining for cytokine production, the cytotoxic assay or p-CREB and p-S6 flow cytometry staining were then performed.

### Cytokine production assay and intracellular staining

Cryopreserved blood mononuclear cells were stimulated with anti-CD3/CD28 beads (bead-to-cell ratio = 1:2; Miltenyi) or CMV−/EBV−/Flu-specific peptides (1 μM). TILs were stimulated by anti-CD3/CD28 beads (bead-to-cell ratio = 1:2; Miltenyi) or autologous tumor cell lines. Stimulation was performed overnight at 37 °C in the presence of GolgiPlug (1 μg/ml; BD) and anti-CD107. When described, cells were preincubated with the A2AR antagonist ZM 241385 (30 μM), the A2BR antagonist PSB 1115 (100 μM), or the PKA inhibitor KT 5720 (30 μM) for 1 h 30 min and then with nothing, Ado (30 μM), or the A2AR agonist CGS 21680 (30 μM) for an additional 2 h. The time of incubation for Ado was chosen as the time giving the lowest reduction in IFN-γ production with no impact on cell survival. The time of incubation of antagonists was chosen as the time needed by ZM 241385 to fully prevent Ado inhibition of IFN-γ production. Then, the cells were washed with PBS-2 mM EDTA and stained extracellularly. First, cells were stained for the surface markers CCR7 and CD45RA and with a viability dye (Zombie UV, Biolegend) for 20 min at 4 °C. Cells were then washed and permeabilized with Cytofix/Cytoperm solution (30 min 4 °C, Fix and Perm buffer, Becton Dickinson), washed with wash buffer (Becton Dickinson) and stained with antibodies directed to CD3, CD4, CD8, IFN-γ, TNF-α and IL-2 (20 min, 4 °C). After washing, the cells were resuspended in PBS and analyzed by *flow cytometry*.

### Functional avidity assessment

The functional avidity of virus-specific CD8^+^ T cell responses was assessed by performing limiting peptide dilutions (ranging from 1 μM to 10 pM) and measuring cytokine production by flow cytometry. The peptide concentration required to achieve a half-maximal IFN-γ response (EC_50_) was determined as described [22]. Peptide stimulation and antibody staining were performed as described in the *cytokine production assay*.

### Functional sensitivity to ado assessment

Functional sensitivity to Ado of peripheral or expanded tumor-infiltrating or peripheral CD8^+^ T cells was assessed by stimulating cells overnight with anti-CD3/anti-CD28-coated beads in the presence of decreasing doses of Ado (ranging from 0 μM to 100 μM) and measuring cytokine production by flow cytometry. The Ado concentration required to achieve a half-maximal cytokine response (IC_50_) was determined. Stimulation and antibody staining were performed as described in the *cytokine production assay*.

### P-CREB^Ser133^, p-S6^Ser235/236^ and p-Akt^Ser473^ flow cytometry staining

Peripheral or expanded tumor-infiltrating CD8^+^ T cells were preincubated with the A2AR antagonist ZM 241385 (30 μM), the A2BR antagonist PSB 1115 (100 μM), or the PKA inhibitor KT 5720 (10 μM) for 1 h and 30 min and then with nothing, Ado (30 μM), or the A2AR agonist CGS 21680 for 2 h (30 μM). Rapamycin (RAPA, 20 μM) was used as a positive control for mTORC1 (p-S6^Ser235/236^) inhibition, and MK2206 (100 nM) was used as a positive control for mTORC2 (p-Akt^Ser473^) inhibition. To evaluate p-S6^Ser235/236^ or p-Akt^Ser473^, CD8^+^ T cells were stimulated for 3 h with anti-CD3/anti-CD28-coated beads or autologous tumor cell lines. Then, the cells were washed with PBS-2 mM EDTA and stained extracellularly. First, cells were stained for the surface markers CCR7, CD45RA and/or CD98, CD71 and with a viability dye (Zombie, Biolegend) for 20 min at 4 °C. Cells were then washed and fixed/permeabilized with the Transcription Factor Staining Buffer Set (eBioscience). Cells were stained with antibodies directed to p-CREB^Ser133^, p-S6^Ser235/236^ and p-Akt^Ser473^ (20 min, 4 °C). p-S6^Ser235/236^ and p-Akt^Ser473^ were not conjugated to a fluorochrome; therefore, after washing, a preadsorbed secondary goat anti-rabbit IgG H&L antibody Alexa Fluor® 488 (Abcam) was added. After washing, the cells were resuspended in PBS and analyzed by flow cytometry.

### Measure of metabolic parameters by flow cytometry

The following assays were performed in glucose-free medium. 2-[N-(7-Nitrobenz-2-oxa-1,3-diazol-4-yl)amino]-2-deoxy-d-glucose (2-NBDG, Invitrogen) uptake, CD98 and CD71 upregulation were carried out with magnetically isolated CD3^+^ T cells (Pan T Cell Isolation Kit, Miltenyi), which were preincubated with ZM 241385 (30 μM), PSB 1115 (100 μM), and KT 5720 (Sigma) for 1 h and 30 min and then with nothing, Ado (30 μM), CGS 21680 or BAY 60–6583 (Sigma) for 2 h and finally activated overnight by anti-CD3/CD28 beads. T cells were then stained with antibodies directed to CD3, CD4, CD8, CCR7, and CD45RA and when needed CD98 and CD71 (20 min 4 °C). To evaluate glucose uptake, cells were washed and incubated with 100 μM 2-NBDG at 37 °C for 20 min prior to fluorescence measurement by *flow cytometry*.

### Detection of AdoR mRNA by RNA flow cytometry (*Prime Flow RNA, Affymetrix*)

Human PBMCs were thawed, and when needed, total CD3^+^ cells were isolated by negative selection with a Pan T Cell Isolation Kit (MACS technology, Miltenyi). Cells were transferred to RNA Flow staining tubes (RNAse free; provided with the kit). Mouse splenocytes were also taken as a negative control and stained for anti-mCD45. Cells were first stained at the surface for CD3, CD4, CD8, CD56, CD16, CD19, CD14, CCR7, and CD45RA (20 min 4 °C) and then stained for AdoR mRNA following the manufacturers’ instructions [23]. Briefly, tubes were centrifuged at 2500 rpm for 5 min. Cells were then fixed and permeabilized in the presence of RNAse inhibitors. A second step of fixation was performed for 1 h at room temperature. Mouse cells were added to each human sample as an internal negative control or in a separate tube. Cells were then washed twice in wash buffer, and target probes for AdoRs were added and incubated for 2 h at 40 °C. After centrifugation, the cells were left overnight at 4 °C. PreAmp-mix and Amp-mix were then added successively with an incubation time of 1 h and 30 min at 40 °C each and two washes between each incubation step. Finally, labeled custom probes directed against *ADORA2A, ADORA2B, ADORA1* and *ADORA3* were added, and the cells were washed before *flow cytometry analysis*.

### Flow cytometry analysis

Flow cytometry acquisition was performed with an LSRFortessa flow cytometer (BD Biosciences). Flow cytometry analysis was performed with FlowJo software (Version 10.2, Treestar). Data were analyzed by Prism v7.

### Preparation of cellular extracts and western blot analysis

Whole-cell extracts were prepared using RIPA buffer (50 mM Tris, 150 mM NaCl, 1% Triton X-100, 1% sodium deoxycholate, 0.1% sodium dodecyl sulfate (SDS), 1 mM phenylmethylsulfonyl fluoride (PMSF), 1 mM Na3VO4, 5 mM NaF, and 1% cocktail protease inhibitors; Sigma). The protein concentration was measured using the Pierce™ BCA Protein Assay Kit (Thermo Scientific). Equal amounts of protein (40 μg/sample) were separated by electrophoresis in a 12% denatured polyacrylamide gel and blotted onto nitrocellulose membranes (Bio-Rad). The membranes were blocked for 1 h in 5% low-fat milk in PBS with 0.1% Tween 20 (PBST) at room temperature. Then, the filters were incubated with the following primary antibodies overnight at 4 °C: Phospho-CREB (Ser133) (9198, Cell Signaling, USA; diluted 1:1000); CREB (9197, Cell Signaling, USA; diluted 1:1000); Phospho-p44/42 MAPK (Erk1/2) (Thr202/Tyr204) (9101, Cell Signaling, USA; diluted 1:1000); p44/42 MAPK (Erk1/2) (9102, Cell Signaling, USA; diluted 1:1000); Phospho-Akt (Ser473) (4058, Cell Signaling, USA; diluted 1:1000); Akt (pan) (2920, Cell Signaling, USA; diluted 1:2000); Phospho-S6 Ribosomal Protein (Ser235/236) (2211, Cell Signaling, USA; diluted 1:1000); S6 Ribosomal Protein (2317, Cell Signaling, USA; diluted 1:1000); and α-Tubulin (3873, Cell Signaling, USA; diluted 1:1000). The membranes were washed 3 times with PBST and then incubated with (HRP)-conjugated anti-rabbit or anti-mouse antibodies for 2 h at room temperature. The membranes were then washed 3 times (10 min in PBST), and proteins were visualized by the ECL chemiluminescence method. The immunoreactive bands of proteins were acquired by using the GBOX Chemi XX6 system (Syngene).

### Cytotoxic assay

When described, TILs were preincubated with the A2AR antagonist ZM 241385 (30 μM) for 1 h and 30 min and then with nothing or Ado for 2 h (30 μM). TILs were then dispensed into a 96-well plate, in triplicate, at decreasing densities: from 100,000 to 1000 cells/well.

Autologous melanoma cell lines or P815 cell lines (unloaded or loaded with anti-CD3) were labelled with ^51^chromium for 45 min at 37 °C, and after 3 washing steps, 1000 cells were dispensed into each TIL-containing well. In addition, for calculation of specific lysis, 1000 cells were also dispensed into four wells containing only tumor medium (spontaneous release) and into four wells containing 1 M HCL (maximum release).

Effector and target cells were incubated in a total volume of 200 μl of RPMI 10% FBS or HCL for 4 h at 37 °C, 5% CO_2_. The plates were centrifuged for 3 min at 230×g. Forty microliters of the culture supernatants was transferred to a LumaPlate-96 (PerkinElmer, Turku, Finland) and, after drying the plates, read on a TopCount NXT (Packard, Meriden, USA). The percentage lysis was calculated by the following formula:

$$ \%\mathrm{of}\ \mathrm{Lysis}=\left[\left(\mathrm{experimental}\ \mathrm{release}-\mathrm{spontaneous}\ \mathrm{release}\right)/\left(\mathrm{maximum}\ \mathrm{release}-\mathrm{spontaneous}\ \mathrm{release}\right)\right]\times 100. $$

### Semiquantitative RT-PCR (qRT-PCR) (SYBR GREEN)

To measure AdoR expression, CD8^+^ T cells were isolated from cryopreserved blood mononuclear cells by magnetic activated cell sorting (MACS technique) using a CD8^+^ T Cell Isolation Kit (Miltenyi Biotech) according to the manufacturer’s instructions. The MCF-7, EBV-B, PC3, THP-1 and HL-60 cell lines were also used as internal controls for validation. AdoR and HIF-1α expression were measured in total TILs. Total cellular RNA was isolated by the RNeasy Micro kit (Qiagen). cDNA was synthesized from 250 ng of total RNA using the Superscript II system according to the manufacturer’s protocol (Life Technologies). PCR reactions contained cDNA, 2x qPCR Master Mix (KAPA Biosystems), and 2 μM of forward and reverse primers. qRT-PCR was performed on an Applied Biosystems® 7500 Fast Real-Time PCR instrument. Each reaction was performed in three replicates, with beta-2-microglobulin (β2M) as an internal control gene for normalization. Raw Ct values were imported into Excel, and the expression levels of the PCR products relative to β2M were calculated using the 2 − ΔCt method.

The primer sequences used were as follows: β2M sense, 5′-CCAGCAGAGAATGGAAAGTC-3′; antisense, 5′-GATGCTGCTTACATGTCTCG-3; *ADORA1* sense, 5′-CCTCCATCTCAGCTTTCCAG-3′ (DOI: 10.1182/blood-2013-02-482406); antisense, 5′-AGTAGGTCTGTGGCCCAATG-3′, *ADORA2A* sense, 5′- CTCCGGTACAATGGCTTGGT-3′; antisense, 5′- TGGTTCTTGCCCTCCTTTGG-3′, *ADORA2B* sense, 5′-ATGCCAACAGCTTGAATGGAT-3′; antisense, 5′-GAGGTCACCTTCCTG GCAAC-3′, ADORA3 sense, 5′-TTGACCAAAAGGAGGAGAAGT-3′; antisense, 5′- AGTCACATCTGTTCAGTAGGA G-3′. HIF-1α sense, 5′-GGC AGC AAC GAC ACA GAA ACT GA-3′; antisense, 5′-TGA TCC TGA ATC TGG GGC ATG GT-3′ (DOI: 10.1038/s41598-017-00508-x).

The efficiency of each primer set was tested by generating a standard curve using relative positive control samples for each primer (MCF-7, EBV-B, PC3 and HL-60 for adenosine receptor A1, A2A, A2B and A3, respectively). All primers were tested by qRT-PCR using a two-fold serial dilution of cDNA and water as a nontemplate control to determine the amplification efficiency and specificity. The amplification efficiency for each primer was determined from the linear slope of the standard curve; only primers with a standard curve slope between − 3.1 and − 3.4 were used for further quantification.

Analysis of the relative changes (arbitrary units) in gene expression required calculations based on the threshold cycle (Ct: the fractional cycle number at which the amount of amplified target reaches a fixed threshold). The threshold cycles of the cell subsets were then normalized based on the threshold cycle of the housekeeping gene (β2M) to obtain the ΔCt.

### Measure of glycolytic and phospho-oxidative metabolism (seahorse assay)

Magnetically isolated CD3^+^ T cells were left unconditioned or incubated with ZM 241385 (30 μM) and/or Ado (30 μM). Cells were then stimulated overnight with anti-CD3/CD28 beads, and the seahorse assay was performed. For the extracellular flux assay, the sensor cartridge was hydrated overnight in Seahorse XF Calibrant at 37 °C in a non-CO_2_ incubator. A total of 4 × 10^5^ human T cells with different treatment conditions were seeded in a Seahorse Bioscience culture plate for 30 min. OCR and ECAR were then measured by an XF96 Seahorse Extracellular Flux Analyzer following the manufacturer’s instructions. During the seahorse assay, cells were treated with oligomycin (0.5 μM), FCCP (2 μM), rotenone (0.5 μM), antimycin A (0.5 μM) and 2-DG (50 mM). Each condition was performed in 3–6 replicates.

### Statistical analysis

Statistical analysis was performed with Prism software (Version 7, GraphPad) using nonparametric and parametric paired (Friedman or Wilcoxon or t test) and unpaired (Kruskal-Wallis or Mann-Whitney) tests as indicated. For multiple comparisons, adjusted *P*-values were calculated by one-way ANOVA followed by Dunn’s test. Correlations were assessed by the nonparametric Spearman’s test.

## Results

### Human CD8^+^ T cell memory subsets are differentially affected by ado

**Fig. 1:**
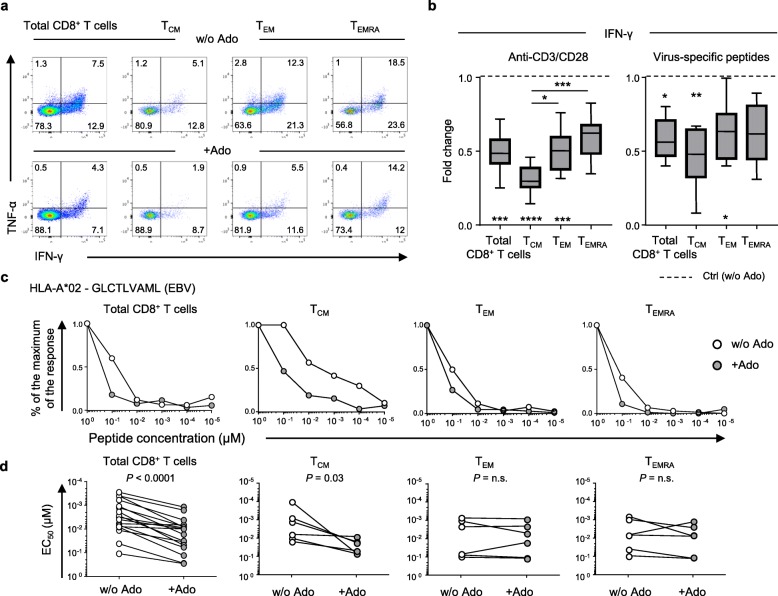
Ado-mediated immunosuppression of T cell function depends on the differentiation stage. **a** Representative example and **b** cumulative data showing the Ado-mediated fold change in IFN-γ production in total and in distinct memory CD8^+^ T cell subsets (T_CM_, T_EM_, and T_EMRA_) stimulated overnight with anti-CD3/anti-CD28-coated beads (*n* = 12) or CMV−/EBV−/Flu-specific peptides *(n* = 11). The 25th to 75th percentiles, the median and min-max of the values are presented. *P < 0.05, **P < 0.01, ***P < 0.001, ****P < 0.0001. Wilcoxon and one-way ANOVA tests. **c** Representative example and **d** cumulative data of the virus-specific CD8^+^ T cell functional avidity measured in the presence or absence of Ado in total (*n* = 12) and in distinct memory CD8^+^ T cell subsets (T_CM_, T_EM_, and T_EMRA_) (*n* = 6). Wilcoxon test

Activated naïve CD8^+^ T cells differentiate in memory subsets with distinct phenotypic and functional properties [24]. The differentiation and maintenance of murine naïve CD8^+^ T cells is negatively impacted by A2AR signaling [25]. However, the differences in the sensitivity to Ado of memory subsets in human CD8^+^ T cells have not yet been explored. We identified the major CD8^+^ T cell memory subsets according to their differential expression of CCR7 and CD45RA (i.e.*,* T central memory-T_CM_, T effector memory-T_EM_, T terminally differentiated-T_EMRA_; Additional file 1: Figure S1a, Additional file 2: Table S1), and we assessed the relative sensitivity to Ado by measuring cytokine production following polyclonal or antigen-specific stimulation (Fig. 1a-b, Additional file 2: Table S1). Exposure to Ado decreased cytokine production (i.e.*,* IFN-γ, TNF-α, and IL-2) and partially decreased the degranulation activity (i.e.*,* CD107a mobilization) in CD8^+^ T cells (Fig. 1a-b and Additional file 1: Figure S1 b-d), consistent with previous studies [14, 16, 17, 20]. Ado mediated suppression of responses to both polyclonal stimulation (i.e.*,* anti-CD3/anti-CD28-coated beads) (Fig. 1a-b and Additional file 1: Figure S1b-d) and viral epitopes (Fig. 1b and Additional file 1: Figure S1c) but not stimulation with ionomycin or phorbol-12-myristate-13-acetate (PMA) (Additional file 1: Figure S1b,e). Of interest, among the distinct T cell subsets, T_CM_ were more sensitive to Ado, with deeper suppression of cytokine production than those of the T_EM_ and T_EMRA_ subsets (Fig. 1b and Additional file 1: Figure S1f).

The functional avidity of CD8^+^ T cells, also called antigen sensitivity, is measured as the peptide concentration able to mobilize 50% of the maximal response (EC_50_). The functional avidity of T cells is independent of the magnitude of a response measured at saturating antigen concentrations [26] and is usually associated with superior control of virus replication or tumor growth [22, 27]. We evaluated the functional avidity of a panel of virus-specific polyclonal CD8^+^ T cells exposed to decreasing peptide concentrations. We observed that Ado significantly reduced the functional avidity of all virus-specific CD8^+^ T cells (Fig. 1c-d), with the strongest effect observed in the T_CM_ subset (Fig. 1c-d). These data indicate that Ado limits the ability of T cells to respond to targets expressing cognate antigens.

### A2AR expression levels dictate ado sensitivity in human CD8^+^ T cells

**Fig. 2:**
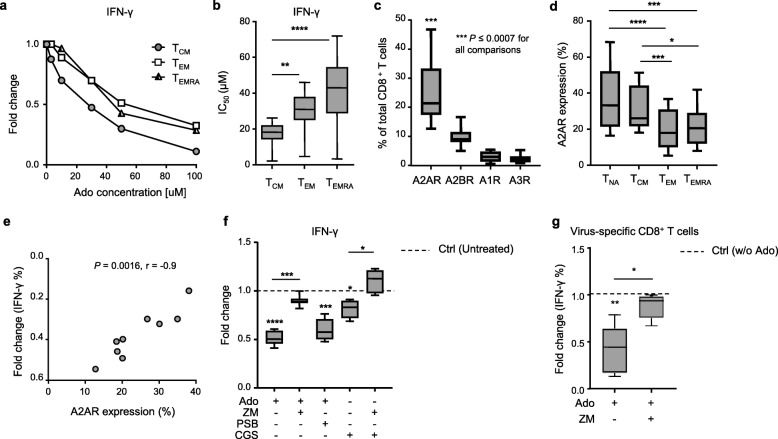
A2AR expression levels are correlated with the extent of Ado-mediated immunosuppression. **a** Representative example and **b** cumulative data of the functional sensitivity (EC_50_ of IFN-γ production) to Ado measured in distinct CD8^+^ T cell memory subsets after overnight stimulation by anti-CD3/CD28 beads in the presence of decreasing concentrations of Ado (*n* = 12). **c** Cumulative data of AdoR expression (i.e.*, ADORA2A* (*n* = 22)*, ADORA2B* (*n* = 22)*, ADORA1* (*n* = 10)*,* and *ADORA3* (*n* = 10)) measured by RNA flow in total CD8^+^ T cells. **d** Cumulative data of A2AR measured by RNA flow in distinct CD8^+^ T cell memory subsets (*n* = 16). **e** Correlation between A2AR expression measured by RNA flow and the Ado-mediated fold change in IFN-γ production in total CD8^+^ T cells. Spearman’s test, *n* = 9. **f** Cumulative data of the fold change in IFN-γ production by CD8^+^ T cells stimulated overnight with anti-CD3/CD28 beads in the presence of the indicated combinations of Ado, A2AR agonist (CGS 21680) and A2AR/A2BR antagonists (ZM 241385 and PSB 1115, respectively). **g** Cumulative data showing the fold change in IFN-γ production by CD8^+^ T cells stimulated overnight with virus-specific peptides in the presence of the indicated combinations of Ado and A2AR antagonist (ZM 241385) (*n* = 6). In all box charts, the 25th to 75th percentiles, the median and min-max of the values are presented; *P < 0.05, **P < 0.01, ***P < 0.001. Wilcoxon and/or one-way ANOVA tests

Our data suggest that T_CM_ cells are the most sensitive subset to functional blunting by Ado. To substantiate this further, we quantified the functional sensitivity to Ado of each memory subset by determining the Ado concentration inhibiting 50% of the maximal response (IC_50_ of the cytokine production). We confirmed that CD8^+^ T_CM_ cells displayed higher sensitivity to Ado than T_EM_ and T_EMRA_ cells (Fig. 2a-b and Additional file 1: Figure S2a).

We therefore hypothesized that the differential sensitivity to Ado may be explained by T_CM_ cells expressing higher AdoR levels than the other memory subsets. Due to the lack of antibodies for flow cytometry analyses for A1R and A3R, we established RNA staining for flow cytometry (*PrimeFlow RNA assay*) [23] (Additional file 1: Figure S2b) to determine the expression of all four ADORs (i.e., *ADORA1, ADORA2A, ADORA2B* and *ADORA3*) in primary human CD8^+^ T cells. Moreover, we used semiquantitative real-time PCR (qRT-PCR) as a validation tool. To include positive controls for each receptor tested, we evaluated AdoR expression in distinct cell lines: EBV-transformed B cell line, human leukemic lines (THP-1 and HL-60), prostate cancer lines (DU-145 and PC-3, LNCaP) and a breast cancer cell line (MCF-7) (Additional file 1: Figure S2c). AdoR expression data obtained from qRT-PCR analysis correlated with flow cytometry values in both cell lines and primary CD8^+^ T cells (Additional file 1: Figure S2d-e). A correlation was also found in CD8^+^ T cells when only A2AR expression was considered (Additional file 1: Figure S2e). Overall, these data indicate that the *PrimeFlow RNA assay* is a suitable technology for the relative quantification of AdoR expression in human CD8^+^ T cells. We also measured A2AR and A2BR expression with antibodies by flow cytometry in CD8^+^ T cells and found a correlation with A2AR expression measured by *PrimeFlow RNA* (Additional file 1: Figure S2f-g). Moreover, we confirmed the lower/undetectable expression of A2BR by CD8^+^ T cells (Additional file 1: Figure S2f, h).

We then evaluated AdoR expression in total CD8^+^ T cells and in different memory subsets ex vivo. Of interest, A2AR was expressed at a higher percentage than other AdoRs in total CD8^+^ T cells (Fig. 2c). Among the memory subsets, it was predominantly expressed by CD8^+^ T_CM_ cells, similar to naïve CD8^+^ T cells (Fig. 2d). These data strongly suggest that the high sensitivity of CD8^+^ T_CM_ cells to Ado is associated with their high expression of A2AR. In addition, our results not only support the role of A2AR as a critical player in Ado-mediated suppressive effects in CD8^+^ T cells but also indicate that A2AR expression quantitatively dictates sensitivity to Ado. This was further corroborated by the positive correlation between A2AR expression in CD8^+^ T cells and the Ado-mediated fold change (i.e.*,* decrease) in IFN-γ production (Fig. 2e), which was not observed for A2BR (Additional file 1: Figure S3a).

Furthermore, we evaluated whether blocking A2AR or A2BR with selective inhibitors (ZM 241385 and PSB 1115, respectively) would prevent the Ado-mediated reduction of cytokine production. The blockade of A2AR, but not A2BR, was able to circumvent the immunosuppressive effects of Ado on CD8^+^ T cells (Fig. 2f). The selective A2AR agonist CGS 21680 was also able to reduce IFN-γ production in CD8^+^ T cells (Fig. 2f). Consistent with what was already shown in the presence of Ado (Fig. 1b), CGS 21680 primarily affected the T_CM_ subset (Additional file 1: Figure S3b). The preventive action of A2AR- or A2BR-selective inhibitors was equal in all the differentiation subsets evaluated (data not shown). The blockade of A2AR or A2BR alone did not affect cytokine production significantly (Additional file 1: Figure S3c). Finally, selective A2AR blockade also efficiently prevented Ado-mediated immunosuppression of virus-specific CD8^+^ T cells (Fig. 2g).

Taken together, these data not only confirm A2A as the main receptor responsible for Ado-mediated immunosuppression but also highlight the relevance of A2AR expression levels in quantitatively determining Ado suppressive effects in primary human CD8^+^ T cells.

### Ado/A2AR triggers PKA, impairing TCR/mTORC1 signaling and metabolic/effector functions in CD8^+^ T cells

**Fig. 3:**
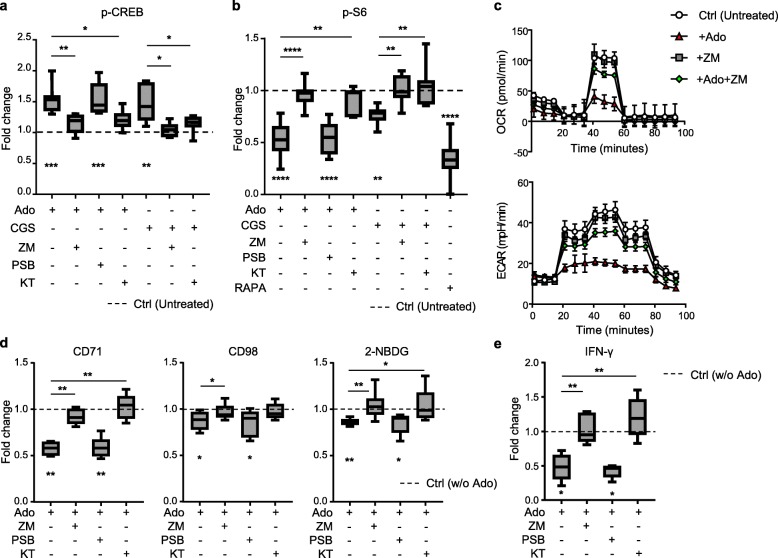
Ado/A2AR signaling modulates PKA and mTORC1 activation and impairs CD8^+^ T cell metabolic fitness and cytokine production. **a** Cumulative data of p-CREB expression in CD8^+^ T cells treated with the indicated combinations of Ado, A2AR agonist (CGS 21680), A2AR/A2BR antagonists (ZM 241385 and PSB 1115, respectively) and the PKA inhibitor KT570 (*n* = 7). **b** Cumulative data of p-S6 expression in CD8^+^ T cells treated with the indicated combinations of Ado, A2AR agonist (CGS 21680), A2AR/A2BR antagonists (ZM 241385 and PSB 1115, respectively), the PKA inhibitor KT570 or the mTOR inhibitor rapamycin (RAPA) and stimulated for 3 h by anti-CD3/anti-CD28-coated beads. **c** Representative example of 3 independent experiments depicting the OXPHOS (measured as the oxygen consumption rate; OCR) and the glycolytic (measured as the extracellular acidification rate; ECAR) metabolism in CD8^+^ T cells stimulated overnight by anti-CD3/CD28-coated beads in the presence of the indicated combinations of Ado and the A2AR antagonist ZM 241385. **d** Cumulative data of CD71 and CD98 expression or 2-NBDG uptake in CD8^+^ T cells treated with the indicated combinations of Ado and A2AR/A2BR antagonists (ZM 241385 and PSB 1115, respectively) or the PKA inhibitor KT570 and stimulated overnight by anti-CD3/anti-CD28-coated beads (*n* = 6, *n* = 7 and *n* = 8 from left to right). **e** Cumulative data of the fold reduction in CD8^+^ T cell IFN-γ production after treatment with combinations of Ado and A2AR antagonist (ZM 241385) or the PKA inhibitor KT570 and stimulated overnight by anti-CD3/anti-CD28-coated beads (*n* = 5). In all box charts, the 25th to 75th percentiles, the median and min-max of the values are presented; *P < 0.05, **P < 0.01, ***P < 0.001. Wilcoxon and/or one-way ANOVA tests

Ado-mediated activation of A2AR increases intracellular cAMP levels [28], and many of the downstream effects of cAMP elevation are dependent upon PKA activation [19, 29]. Of interest, the selective inhibition of the PKA pathway in CD8^+^ T cell clones can prevent Ado-induced suppression of cytokine production [20]. We therefore investigated whether molecular mediators of PKA signaling were activated by Ado in primary human CD8^+^ T cells. Basal p-CREB levels increased in the presence of Ado or CGS 21680, which is a selective A2AR agonist (Fig. 3a and Additional file 1: Figure S4a-b). This effect was prevented by both the PKA-specific inhibitor KT 5720 (Fig. 3a and Additional file 1: Figure S4a-b) and the selective A2AR antagonist ZM 241385 (Fig. 3a and Additional file 1: Figure S4a-b), while it was not affected by the selective A2BR antagonist (PSB 1115, Fig. 3a). All memory T cell subsets were influenced by Ado (Additional file 1: Figure S4c), while CGS 21680 mostly affected the T_CM_ subset (Additional file 1: Figure S4c), mirroring the functional data (Fig. 1).

Consistent with the increase in PKA activation and previous observations [21, 30], we observed that Ado reduces TCR-dependent ERK phosphorylation in primary human CD8^+^ T cells, reflecting impaired TCR signaling. Notably, this effect was dependent upon A2AR/PKA signaling (Additional file 1: Figure S4d-e).

Since the mTOR pathway is pivotal for complete T cell activation and induces potent effector functions [4, 31, 32], we evaluated whether Ado/A2AR signaling could affect mTOR triggering in a PKA-dependent manner. To this end, we assessed the phosphorylation of downstream effectors of mTORC1 and mTORC2, i.e.*,* S6 (p-S6; Additional file 1: Figure S5a-b) and Akt^Ser473^ (p-Akt^Ser473^; Additional file 1: Figure S5c-d) after polyclonal CD8^+^ T cell stimulation. Ado signaling markedly impaired phosphorylation of S6 (Fig. 3b and Additional file 1: Figure S5a-b) but not of Akt^Ser473^ (Additional file 1: Figure S5c-e). These data indicate that the mTORC1, but not the mTORC2, complex is a target of Ado-mediated signaling in primary human CD8^+^ T cells. Importantly, this effect was mediated by A2AR activation since the selective A2AR agonist CGS 21680 also reduced S6 phosphorylation (Fig. 3b), and the selective inhibitor of A2AR (ZM 241385), but not A2BR (PSB 1115), was able to prevent both Ado and CGS 21680 effects (Fig. 3b). Interestingly, S6 phosphorylation was reduced in both T_CM_ and T_EM_ subsets but not in the T_EMRA_ subset (Additional file 1: Figure S5f). Additionally, the selective agonist of A2AR (CGS 21680) predominantly affected the T_CM_ compartment (Additional file 1: Figure S5f), mirroring the functional data (Fig. 1). Finally, blockade of the PKA pathway prevented Ado-mediated inhibition of mTORC1 activation (Fig. 3b), thus indicating that Ado impaired mTORC1 signaling through PKA activation. These data strongly suggest that Ado/A2AR signaling impairs mTORC1 activation in human CD8^+^ T cells through PKA, likely due to the PKA-dependent inhibition of TCR signaling.

mTOR is the main complex responsible for both metabolic rewiring [3, 33] and augmented effector functions following TCR-mediated activation [34, 35]; thus, we assessed whether Ado also impaired CD8^+^ T cell metabolic fitness. Upon CD8^+^ T cell activation, Ado impaired metabolic activity of both OXPHOS and glycolysis in an A2AR-dependent manner (Fig. 3c). However, Ado induces more severe impairment of ECAR, suggesting that cells prefer to utilize oxygen consumption to sustain metabolic needs in this condition.

This was further confirmed by the Ado-mediated impairment of CD71/CD98 expression and of 2-NBDG (glucose analog) uptake (Fig. 3d and Additional file 1: Figure S6a). The selective inhibition of A2AR, but not of A2BR, prevented the Ado-mediated effects (Fig. 3d). Among the memory CD8^+^ T cell subsets, T_CM_ cells were the most affected (Additional file 1: Figure S6b). Notably, both PKA and A2AR inhibition were able to prevent Ado-mediated impairment of metabolic fitness and cytokine production (Fig. 3d-e).

Taken together, these results strongly suggest that the Ado/A2AR-mediated increase in PKA activation in primary human CD8^+^ T cells leads to subsequent impairment in metabolic fitness and effector functions, likely due to the reduction in TCR signaling and mTORC1 activation.

### Ado suppresses TILs activation and tumor recognition

**Fig. 4:**
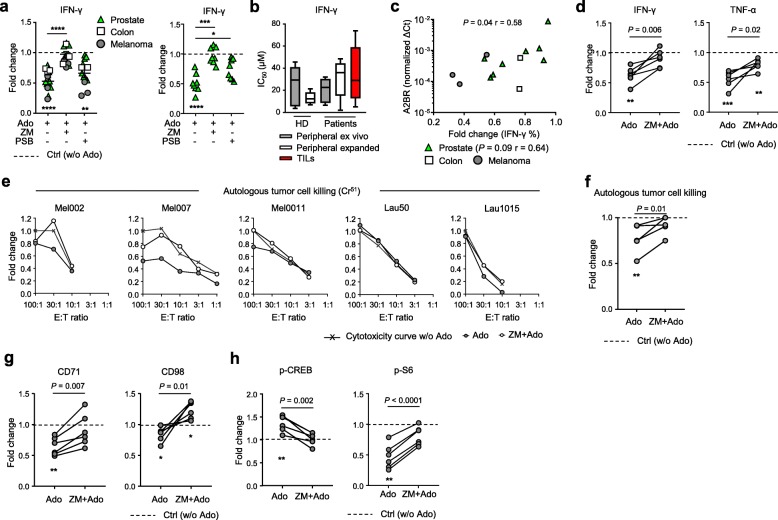
Ado impairs TIL effector functions and autologous tumor cell recognition. **a** Cumulative data of the fold change in CD8^+^ TIL IFN-γ production in the presence of the indicated combinations of Ado, A2AR agonist (CGS 21680) and A2AR/A2BR antagonists (ZM 241385 and PSB 1115, respectively) (*n* = 14). The left graph shows all the TIL samples analyzed, while the right graph only shows prostate-derived samples. **b** Cumulative data of the functional sensitivity (IC_50_ of IFN-γ production) to Ado measured in resting or expanded peripheral CD8^+^ T cells (*n* = 5) derived from HD or patients and in CD8^+^ TILs (*n* = 10) derived from patient tissues. The functional sensitivity was measured after overnight stimulation by anti-CD3/CD28-coated beads in the presence of decreasing concentrations of Ado. **c** Correlation between A2BR expression measured by qRT-PCR and the Ado-mediated fold change in IFN-γ production in CD8^+^ TILs. Spearman’s test, *n* = 13 (*n* = 8 for the prostate sample analysis). **d** Cumulative data of the fold change in IFN-γ and TNF-α production by TILs stimulated overnight by autologous tumor cells in the presence of Ado or ZM 241385 + Ado. (*n* = 6) **e** Each graph represents cytotoxicity curves for one patient quantified in the absence of Ado, in the presence of Ado or ZM 241385 + Ado. Data are presented as normalized to the cytotoxicity measured in the absence of Ado at the effector:target (E:T) ratio of 100:1. Cytotoxicity was measured by coincubating TILs for 4 h with autologous tumor cells; measurements were performed in triplicate. **f** Cumulative data of the fold change in cytotoxicity in the presence of Ado or ZM 241385 + Ado (*n* = 5). **g** Cumulative data of the fold change in CD71 and CD98 expression by TILs stimulated overnight by autologous tumor cells in the presence of Ado or ZM 241385 + Ado (*n* = 6). **h** Cumulative data of the fold change in p-CREB and p-S6 expression by TILs stimulated for 3 h by autologous tumor cells in the presence of Ado or ZM 241385 + Ado (*n* = 6). In all charts, each dot represents a patient, and the mean and standard error are presented. In all box charts, the 25th to 75th percentiles, the median and min-max of the values are presented; *P < 0.05, **P < 0.01, ***P < 0.001. Paired t test and/or one-way ANOVA tests

Ado is a mediator of TME immunosuppression and may limit the success of immunotherapy, notably the adoptive cell transfer of tumor infiltrating lymphocytes (TILs) [13, 36]. Since little is known regarding the susceptibility of human TILs to Ado-mediated suppression and the Ado receptors involved [16], we evaluated the capacity of selective A2AR and A2BR inhibitors to prevent Ado immunosuppression in human TILs. TILs were expanded with the REP (rapid expansion protocol) from tumoral or inflamed/normal prostate (*n* = 8), colon cancer metastasis (*n* = 4) and metastatic melanoma (*n* = 6) (Additional file 2: Table S2); cell cultures with heterogeneous CD4^+^/CD8^+^ T cell ratios were obtained according to the tumor evaluated (Additional file 1: Figure S7a). Although heterogeneous (Additional file 1: Figure S7b), the cytokine production capacity of CD8^+^ TILs was consistently suppressed by Ado (Additional file 1: Figure S7c). In line with the effects of Ado on peripheral T cells, inhibitors of A2AR prevented the Ado-mediated suppression of CD8^+^ TILs (Fig. 4a and Additional file 1: Figure S7c). Surprisingly, in prostate-derived samples, A2BR blockade was also able to prevent the Ado effect (Fig. 4a and Additional file 1: Figure S7c). However, we did not find any impact of A2AR or A2BR inhibitors on the TIL expansion capacity (data not shown). We also evaluated the sensitivity of CD8^+^ TILs to Ado and compared it to circulating CD8^+^ T cells from healthy donors and cancer patients in resting conditions (ex vivo) or after in vitro expansion. Despite high variability, we observed that CD8^+^ TILs and circulating CD8^+^ T cells had an overall similar sensitivity (IC_50_) to Ado (Fig. 4b). To gain further insight into the AdoR responsible for Ado-mediated immunosuppression in TILs, we evaluated AdoR expression by qRT-PCR in total TIL products. As for the REP-expanded healthy peripheral T cells, TILs predominantly expressed A2AR and lacked A1R and A3R (Additional file 1: Figure S7d). However, TILs showed higher expression of A2BR in some cases, in particular the ones derived from prostate tissues (Additional file 1: Figure S7d). Notably, we found a correlation between A2BR expression and the capacity of the A2BR antagonist to prevent the Ado-mediated reduction in IFN-γ production **(**Fig. 4c). The higher expression of A2BR can only be partially explained by the T cell activation status. Indeed, CD8^+^ T cells stimulated moderately and transiently upregulated A2BR in vitro at day 3 (Additional file 1: Figure S7e).

Previous data support the hypothesis that hypoxia could induce the activation of the CD73-A2AR adenosine pathway and that A2AR expression was positively correlated in head and neck squamous cell carcinoma tissues [18, 37, 38]. However, we did not find a correlation between the expression of HIF-1α and A2AR in the expanded TILs (Additional file 1: Figure S7f).

Ado can inhibit the ability of TILs to recognize and kill target cells loaded with the melanoma differentiation antigen MART1_25–36_ peptide [39]. However, the effect of Ado on autologous tumor cell recognition was not addressed. We first characterized AdoR expression in 6 autologous melanoma cell lines (Additional file 2: Table S2) by qRT-PCR and compared it to AdoR expression in TILs expanded from the same lesion. In contrast to TILs, tumor cells expressed A2AR at lower levels than A2BR and at levels comparable to A1R and A3R (Additional file 1: Figure S8a). We then evaluated whether Ado could impair the ability of TILs to respond to autologous tumor cells in vitro. Of interest, Ado was able to reduce cytokine production, cytotoxic activity and metabolic fitness of melanoma-derived TILs cocultured with autologous tumor cells (Fig. 4d-g and Additional file 1: Figure S8b-c). In the redirected killing assay, the maximum frequency of cell lysis was 100% for all patients tested, and the suppressive effect of Ado on cytotoxic function was almost undetectable (less than 20% reduction for any ratio considered) (Additional file 1: Figure S8d). In contrast, in the autologous setting, Ado inhibition was more pronounced, reaching a 50% reduction for some of the ratios tested where the maximum level of cell lysis was approximately 20% (Mel002, Mel007, and Lau1015) (Fig. 4e). The lowest effect was observed for patients Mel0011 and Lau50, where the cytotoxicity was naturally very high (91 and 100%). We could not test the effect of Ado in patient Lau1660 due to a low overall killing capacity (less than 10%). In the autologous setting, when the highest lysis values were considered (effector:target ratio E:T = 100:1), a significant reduction of cytotoxicity was observed in the presence of Ado, which was prevented by A2AR blockade (ZM 241385) (Fig. 4f). These observations suggest that in a physiological setting where tumor rejection antigens may be poorly expressed by tumors or when TILs contain limited frequencies of tumor antigen-specific T cells, then Ado may play an important role in preventing tumor cell killing by activating A2AR signaling. The variability in the Ado effect observed at different E:T ratios, however, indicates that further investigation is needed to confirm this evidence. Notably, Ado-mediated immunosuppression in the autologous setting was concomitant with a decrease in metabolic fitness (i.e. CD71 and CD98 upregulation, Fig. 4g), an increase in p-CREB and a decrease in p-S6 activation (Fig. 4h and Additional file 1: Figure S8e-f); these effects were also prevented by the selective inhibition of A2AR (Fig. 4d-h). These data suggest a predominant role of Ado in mediating the suppression of human expanded TILs through A2AR signaling, thus potentially jeopardizing the efficacy of adoptive cell transfer.

## Discussion

Several targets, such as CD39, CD73 and AdoRs, contributing to the accumulation of Ado and its immunosuppressive effects in the TME are under evaluation in preclinical and clinical studies of cancer immunotherapy [40–43]. Ado is known to suppress tumor immunity by reducing immune cell infiltration, cytotoxicity and cytokine production [20, 43]. Moreover, CD8^+^ T cell effector functions and metabolic fitness are dramatically impaired in the TME [9, 10]; thus, understanding the mechanisms of action of immunosuppressive molecules is pivotal to restoring an efficient anti-tumor immune response. However, mediators of Ado signaling are not fully understood, and biomarkers of efficacy for therapies targeting the adenosinergic pathway remain unknown. Our study unveils new mechanisms of Ado-mediated suppression in CD8^+^ T cells and highlights potential new biomarkers to monitor during Ado-targeted immunotherapies.

The inhibition of T cell activation in early-differentiated cells can preclude the regeneration of the effector cells that will die after exerting their cytotoxic/inflammatory function. T_CM_ cells have a longer half-life than effector cells and an enhanced ability to divide and differentiate into more differentiated subsets [24]. We found that human CD8^+^ T_CM_ cells are the most sensitive to Ado due to their high expression of A2AR. A high concentration of Ado in the TME may therefore negatively affect the renewal of fully differentiated effector cells. Studies in both mice and, to a lesser extent, humans support the main role of A2AR in mediating Ado-dependent T cell immunosuppression and exhaustion in the TME [16, 17, 20, 21]. We identified A2AR as the main mediator of the Ado immunosuppressive effect in peripheral and tumor-infiltrating human CD8^+^ T cells, strengthening the importance of this target in next-generation immunotherapy. As previously suggested [20, 21], we confirmed that the Ado/A2AR-mediated suppression in human CD8^+^ T cells relies on PKA activation and inhibition of TCR signaling. To evaluate the expression of the 4 AdoRs, we used RNA flow technology due to the lack of anti-A1R and anti-A3R antibodies available for flow cytometry. We found consistent expression of A2A and A2B receptors measured by RNA flow and by antibodies. However, these antibodies are not optimal for flow cytometry since both were developed for western blotting; anti-A2AR is against an intracellular epitope, thus requiring permeabilization of the cells and not allowing the distinction between the surface and the intracellular compartment, while the anti-A2BR is polyclonal, thus affecting his affinity and specificity. Finally, these antibodies cannot be combined in the same panel since A2BR staining is erased by cell permeabilization.

Independent studies have reported the Ado inhibitory effect on the mTORC pathway and chemotaxis in neutrophils [44] and the presence of a PKA-dependent manner of mTORC1 inhibition in nonimmune cells that was unrelated to the classical mTORC1 activators (i.e.*,* TSC2, Rheb and Rag GTPases) [45, 46]. However, there is no previous evidence demonstrating that Ado impairs mTORC1 activation in an A2AR/PKA-dependent manner in CD8^+^ T cells. Herein, we demonstrate that Ado reduces TCR-dependent mTORC1 activation by triggering A2AR and PKA. Although mTORC1-independent effects cannot be excluded, the importance of this complex in mediating effective T cell activation [1, 4, 31, 32] strongly suggests that, among the targets of TCR signaling, impairment of mTORC1 activation is responsible for most of the Ado/A2AR/PKA-mediated suppressive effects in human CD8^+^ T cells. Notably, we show that Ado signaling in both peripheral CD8^+^ T cells and TILs exposed to autologous tumors or to polyclonal stimulation not only impaired cytokine production but also metabolic fitness. The impairment in mTORC1 activation is presumably the leading cause of the impairment in CD8^+^ T cell metabolism. From a translational standpoint, this evidence implies that Ado/A2AR-mediated immunosuppression may be reverted as well by molecules aiming at improving mTOR activation and metabolism selectively in T cells [47, 48]. On the other hand, the Ado pathway may be key in leading to T cell metabolism uncoupling in the TME, and thus, targeting this signaling may restore the metabolic fitness of infiltrating T cells.

We also characterized AdoR expression in melanoma-derived tumor cell lines and in expanded TILs. Tumor cell lines show higher expression of A2BR than A2AR, possibly reflecting their metastatic origin [49]. Expanded TILs, despite a minor increase in A2BR expression, show a prevalent and almost exclusive expression of A2AR along with results obtained in peripheral CD8^+^ T cells. In line with this evidence, we show that the sensitivity to Ado of CD8^+^ TILs derived from three different tumor types is not different from that of peripheral CD8^+^ T cells. However, TILs derived from prostate samples also express variable amounts of A2BR, and the selective inhibition of this receptor prevents Ado-mediated immunosuppression. This effect was quantitatively correlated with the level of A2BR expression. Although further investigation is needed, the monitoring of A2BR expression in TIL products may be of interest for next-generation personalized immunotherapy in the context of adoptive cell transfer.

Despite indications in the literature of a causal connection between hypoxia and the adenosine pathway, we did not find a correlation between the expression of HIF-1α and A2AR. This may be explained by the lack of a hypoxic environment in the in vitro TIL culture that is instead present in tumor tissues.

Remarkably, Ado is able to affect both polyclonal responses of TILs derived from multiple tumors and autologous tumor-cell recognition by melanoma TILs by reducing cytokine production and metabolic fitness. Ado is therefore able to exert systemic immune suppression as well as to impair antitumor activity. Along the same lines, we show a reduction in the killing capacity of autologous targets at some E:T ratios. This effect was barely observed in the redirected killing assay where killing was 100%. In vivo, however, tumor antigens are not expressed homogeneously throughout the tumor tissue, and their accessibility is limited to specific T cells. Thus, in this context, Ado may exert an inhibitory effect on killing capacity. Our data support this possibility; however, since the impairment in the in vitro cytotoxicity observed is dependent on the E:T ratio, further investigations are needed to explore this hypothesis. The blockade of A2AR abrogated Ado-mediated immunosuppression by preventing an increase in p-CREB and by restoring p-S6 activity. Finally, we did not find any effect of A2AR or A2BR inhibitors on the TIL expansion capacity. This may be due to the absence of a significant concentration of Ado in the culture or to the instability of the compound used that would not allow for observing a significant effect in long-term cultures. This should be better investigated by using drugs currently under investigation in clinical trials [50]. Nevertheless, the AdoR expression and the susceptibility of expanded TILs to Ado immunosuppression described in our study strongly support the Ado/A2AR pathway as an important target for cancer immunotherapy with particular relevance for combinatorial T cell-based strategies.

The recent remarkable clinical success of immunotherapy brings along the need to discover predictive biomarkers of treatment efficacy and related adverse effects. This will help immune oncologists make decisions before and during therapy. To date, judgements are mainly based on the overall assessment of clinical improvement and treatment tolerance; however, early immunomodulation markers need to be unveiled to act faster and more specifically to improve clinical success rates. In this context, we observed that A2AR expression levels quantitatively dictate CD8^+^ T cell sensitivity to Ado and that A2AR blockade rapidly affected p-CREB and p-S6 levels during autologous tumor cell recognition. Therefore, in future preclinical or clinical studies testing therapies targeting the adenosinergic pathway, it will be relevant to monitor T cell expression of A2AR to predict their sensitivity to Ado but also of p-CREB and p-S6 to unveil their potential as biomarkers.

## Conclusions

Overall, our data support the relevance of targeting the Ado/A2AR immunosuppressive pathway to restore both effector function and metabolic fitness of peripheral and tumor-derived CD8^+^ T cells. This could be of particular interest in combination with adoptive cell transfer to prevent TME immunosuppression and improve treatment efficacy. Finally, we postulate that the modulation of A2AR, p-CREB and p-S6 expression levels may be valuable efficacy biomarkers of A2AR blockade and T cell function recovery in the context of clinical studies.

## Additional files

Additional file 1: **Figure S1.** Effects of Ado on CD8^+^ T cell cytokine production capacity. (**a**) Representative example of CD8^+^ T cell differentiation subsets identification by flow cytometry. (**b**) Representative example of cytokine production (i.e. IFN-γ, TNF-α, IL-2 and CD107) by CD8^+^ T cells stimulated overnight with anti-CD3/anti-CD28 coated beads or PMA/Ionomycin in presence or not of Ado. (**c**) Cumulative data showing the fold change in cytokine production (IL-2 and TNF-α) and CD107 expression by CD8^+^ T cells stimulated overnight with virus-specific peptides (*n* = 11) or anti-CD3/anti-CD28 coated beads (*n* = 12) in unconditioned media or in presence of Ado. The 25th to 75th percentiles, the median and min-max of the values are represented. ***P < 0.001, ****P < 0.0001, one-way ANOVA test. (**d**) Cumulative data showing the frequency of cytokine production (IL-2 and TNF-α) and CD107 expression by CD8^+^ T cells stimulated overnight with anti-CD3/anti-CD28 coated beads in unconditioned media or in presence of Ado. The 25th to 75th percentiles, the median and min-max of the values are represented; *n* = 12. *P < 0.05, **P < 0.01, Wilcoxon test. (**e**) Cumulative data showing the fold change in IFN-γ production by CD8^+^ T cells stimulated overnight with anti-CD3/anti-CD28 coated beads or PMA/Ionomycin in presence of Ado. The 25th to 75th percentiles, the median and min-max of the values are represented; *n* = 7. ***P < 0.001, one-way ANOVA test. (**f**) Cumulative data of the fold change in cytokine production (IL-2 and TNF-α) and CD107 expression after overnight stimulation with anti-CD3/anti-CD28 coated beads in presence of Ado in distinct memory CD8^+^ T-cell subsets (T_CM_, T_EM_, T_EMRA_). The 25th to 75th percentiles, the median and min-max of the values are represented; *n* = 12. *P < 0.05, ****P < 0.0001, one-way ANOVA test. **Figure S2.** Effects of Ado on CD8^+^ T cell functional avidity and evaluation of AdoR expression. (**a**) Cumulative data of the functional sensitivity (IC_50_ of IL-2 and TNF- α production) to Ado measured in distinct CD8^+^ T cell memory subsets after overnight stimulation with anti-CD3/CD28 beads in presence of decreasing concentrations of Ado. The 25th to 75th percentiles, the median and min-max of the values are represented; *n* = 12. *P < 0.05, ****P < 0.0001, one-way ANOVA test. (**b**) Representative example of AdoR expression measured by RNA flow in total CD8 T cells. (**c**) Cumulative data of the AdoR expression in EBV transformed B cell line, THP-1, HL-60, PC-3, LNCAP, DU145, MCF-7 measured by qRT-PCR. (**d-e**) Correlation between AdoR expression measured by RNA flow and qRT-PCR in (**d**) cell lines and (**e**) primary human CD8 T cells. Spearman tests. (**f**) Represenative example of flow cytometry staining of total CD8 T cells by anti-A2AR and anti-A2BR antibodies. (**g**) Correlation between the expression of A2AR measured by antibody staining and RNA staining for flow cytometry in total CD8^+^ T cells. Spearman test. (**h**). Cumulative data of the expression of A2AR and A2BR in total CD8^+^ T cells measured by antibody staining for flow cytometry. The 25th to 75th percentiles, the median and min-max of the values are represented; *n* = 9. **Figure S3.** Functional consequences of AdoRs signaling. (**a**) Correlation between A2BR expression measured by RNA flow and the Ado-mediated fold change in IFN-γ production evaluated in total CD8^+^ T cells. Spearman test, *n* = 9. (**b**) Cumulative data showing the fold change in IFN-γ production by distinct memory CD8^+^ T cell subsets (T_CM_, T_EM_, T_EMRA_) stimulated with anti-CD3/anti-CD28 coated beads in presence of the A2AR selective agonist CGS 21680. The 25th to 75th percentiles, the median and min-max of the values are represented; *n* = 6. **P < 0.01, ***P < 0.001, one-way ANOVA test. (**c**) Cumulative data showing the fold change in IFN-γ production by CD8^+^ T cells stimulated with anti-CD3/anti-CD28 coated beads in presence of Ado alone, or the A2AR selective antagonist (ZM 241385) alone, or the A2BR selective antagonist (PSB 1115) alone. The 25th to 75th percentiles, the median and min-max of the values are represented; *n* = 6. ****P < 0.0001, one-way ANOVA test. **Figure S4.** Ado/A2AR impact p-CREB and TCR signaling activation. (**a**) Representative example of p-CREB expression detected by flow cytometry in total CD8^+^ T cells after treatment with Ado or the indicated combination of A2AR agonist (CGS 21680) and antagonist (ZM 241385) or the PKA inhibitor (KT570). (**b**) Representative western blot analysis of p-CREB and CREB in total CD8^+^ T whole-cell lysates after treatment with Ado or the depicted combination of A2AR antagonist (ZM 241385) or the PKA inhibitor (KT570). α-Tubulin was detected as a loading control. *n* = 3. (**c**) Cumulative data showing the fold change in p-CREB expression by distinct memory CD8^+^ T cell subsets (T_CM_, T_EM_, T_EMRA_) in presence of Ado or the A2AR selective agonist CGS 21680. The 25th to 75th percentiles, the median and min-max of the values are represented; *n* = 7. *P < 0.05, **P < 0.01, ***P < 0.001, one-way ANOVA test. (**d**) Representative western blot analysis and (**e**) cumulative data of p-ERK and ERK in total CD8^+^ T whole-cell lysates after treatment with Ado and stimulated for 3 h by anti-CD3/anti-CD28 coated beads or the depicted combination of A2AR antagonist (ZM 241385) or the PKA inhibitor (KT570). α-Tubulin was detected as a loading control. *n* = 3. **Figure S5.** Ado/A2AR impact p-S6 activation. The data represented in this figure were obtained analyzing CD8^+^ T cells stimulated for 3h by anti-CD3/anti-CD28 coated beads under the distinct described conditions. (**a**) Representative example of p-S6 expression detected by flow cytometry after treatment with Ado or the depicted combination of A2AR agonist (CGS 21680) and A2AR/A2BR antagonists (ZM 241385 and PSB 1115). (**b**) Representative western blot analysis of p-S6 and S6 after treatment with Ado alone or combined with the A2AR antagonist (ZM 241385) or the PKA inhibitor (KT570), Rapamycin, the AKT1/2 inhibitor (MK2206). α-Tubulin was detected as a loading control. *n* = 3. (**c**) Representative example of p-Akt^Ser473^ expression detected by flow cytometry in total CD8^+^ T cells stimulated for 3 h by anti-CD3/anti-CD28 coated beads in resting condition or after treatment with Ado. (**d**) Representative western blot analysis of p-Akt^Ser473^ and Akt^Ser473^ after treatment with Ado alone or combined with the A2AR antagonist (ZM 241385) or the PKA inhibitor (KT570), Rapamycin, the AKT1/2 inhibitor (MK2206) and stimulated for 3 h by anti-CD3/anti-CD28 coated beads. α-Tubulin was detected as a loading control. *n* = 3. (**e**) Cumulative data showing the fold change in p-Akt^Ser473^ expression expression after treated with the indicated combinations of Ado, A2AR agonist (CGS 21680), A2AR/A2BR antagonists (ZM 241385 and PSB 1115, respectively) or the AKT_1/2_ inhibitor (MK2206). The 25th to 75th percentiles, the median and min-max of the values are represented; *n* = 7. (**f**) Cumulative data showing the fold change in p-S6 expression by distinct memory CD8^+^ T cell subsets (T_CM_, T_EM_, T_EMRA_) in presence of Ado or the A2AR selective agonist CGS 21680. The 25th to 75th percentiles, the median and min-max of the values are represented; *n* = 14 and *n* = 12 from left to right. **P < 0.01, ***P < 0.001, ****P < 0.0001, one-way ANOVA test. **Figure S6.** Ado affects CD8^+^ T cell glycolytic metabolism. (**a**) Representative example of CD71, CD98 expression and 2-NBDG uptake detected by flow cytometry in total CD8^+^ T cells in resting condition or after treatment with Ado and stimulated overnight by anti-CD3/anti-CD28 coated beads. (**b**) Cumulative data showing the fold change of CD71, CD98 expression and 2-NBDG uptake by distinct memory CD8^+^ T cell subsets (T_CM_, T_EM_, T_EMRA_) stimulated overnight by anti-CD3/anti-CD28 coated beads in resting condition or after treatment with Ado. The 25th to 75th percentiles, the median and min-max of the values are represented; *n* = 17, *n* = 11, *n* = 14 from left to right. **P < 0.01, ***P < 0.001, ****P < 0.0001, one-way ANOVA test. **Figure S7.** Composition, functionality and AdoR expression in TILs. (**a**) Proportion of CD4^+^ and CD8^+^ T cells in REP expanded TILs derived from normal/inflamed or tumor prostate tissue and metastasis of colon cancer or of melanoma. (**b**) Cumulative data of the frequency of cytokine-producing or CD107^+^ CD8^+^ TILs. Each dot represents a patient, lines indicate Mean ± SEM; *n* = 1. (**c**) Cumulative data of the fold change in IL-2, TNF-α production and CD107 expression by TILs stimulated overnight with autologous tumor cells in presence of Ado or ZM 241385 + Ado or PSB 1115 + Ado. Each dot represents a patient, lines indicate Mean ± SEM; *n* = 14. **P < 0.01, ***P < 0.001, ****P < 0.0001, ****P < 0.0001, one-way ANOVA test. (**d**) Cumulative data of the expression of AdoR in peripheral T cells from healthy donors and in TILs derived from the depicted tissue types. The 25th to 75th percentiles, the median and min-max of the values are represented by boxes. Each dot represents a patient. The positivity threshold is determined as 2 log higher expression than values given by water. (**e**) Cumulative data of the expression of AdoR in peripheral CD8^+^ T cells from healthy donors at day 0 or after 3 and 10 days stimulation with anti-CD3/anti-CD28 coated beads. Each dot represents the mean of 8 healthy donors analyzed. *P < 0.05, one-way ANOVA test. (**f**) Correlation between the A2AR and HIF-1α expression measured by qRT-PCR in TILs. Spearman test, *n* = 13. **Figure S8.** AdoR expression and Ado immunosuppression in TILs/autologous tumor cells setting. (**a**) Cumulative data of the expression of AdoR in peripheral melanoma-derived TILs and tumor cells. The 25th to 75th percentiles, the median and min-max of the values are represented by boxes; *n* = 6. (**b**) Representative example of cytokine production in melanoma-derived TILs after overnight stimulation with autologous tumor cells in untreated culture condition, in presence of Ado or ZM 241385 + Ado. (**c**) Representative example of CD71 and CD98 expression by TILs stimulated for overnight by autologous tumor cells in resting condition, in presence of Ado or ZM 241385 + Ado. (**d**) Each graph represents cytotoxicity curves for one patient quantified in resting condition, in presence of Ado or ZM 241385 + Ado. Data are represented as normalized to the cytotoxicity measured in absence of Ado at the effector:target (E:T) ratio 100:1. Cytotoxicity was measured co-incubating TILs for 4 h with P815 cell line loaded with anti-CD3 (redirected killing; bottom graphs); *n* = 3, measures were performed in triplicates. (**e**) Representative example of p-CREB expression by TILs in untreated culture condition or in presence of Ado or ZM 241385 + Ado. (**f**) Representative example of p-S6 and CD107 expression by TILs stimulated for 3 h by autologous tumor cells in untreated culture condition or in presence of Ado or ZM 241385 + Ado. (ZIP 521 kb)

Additional file 2: **Table S1.** Distribution of memory subsets in total and virus-specific CD8 + T cells. **Table S2:** Clinical characteristics of the patients. (ZIP 61 kb)

## Data Availability

The data generated and/or analyzed during the current study are available upon reasonable request to the corresponding author.

## Abbreviations

Ado: Adenosine
AdoR: Adenosine receptor
E:T: Effect:target (ratio)
PBMCs: Peripheral blood mononuclear cells
PKA: Protein kinase A
PMA: Phorbol 12-myristate 13-acetate
qRT-PCR: Semiquantitative real-time PCR
REP: Rapid expansion protocol
T_CM_: T central memory cells
TCR: T cell receptor
TILs: Tumor-infiltrating lymphocytes
TME: Tumor microenvironment
