# Supplementary material for: Adenosine mediates functional and metabolic suppression of peripheral and tumor-infiltrating CD8+ T cells
Source: J Immunother Cancer. 2019 Oct 10;7:257. doi: 10.1186/s40425-019-0719-5 (PMC6788118; doi:10.1186/s40425-019-0719-5)
Supplement: Supplementary file 1 — Figure S1. Effects of Ado on CD8+ T cell cytokine production capacity. (a) Representative example of CD8+ T cell differentiation subsets identification by flow cytometry. (b) Representative example of cytokine production (i.e. IFN-γ, TNF-α, IL-2 and CD107) by CD8+ T cells stimulated overnight with anti-CD3/anti-CD28 coated beads or PMA/Ionomycin in presence or not of Ado. (c) Cumulative data showing the fold change in cytokine production (IL-2 and TNF-α) and CD107 expression by CD8+ T cells stimulated overnight with virus-specific peptides (n = 11) or anti-CD3/anti-CD28 coated beads (n = 12) in unconditioned media or in presence of Ado. The 25th to 75th percentiles, the median and min-max of the values are represented. ***P < 0.001, ****P < 0.0001, one-way ANOVA test. (d) Cumulative data showing the frequency of cytokine production (IL-2 and TNF-α) and CD107 expression by CD8+ T cells stimulated overnight with anti-CD3/anti-CD28 coated beads in unconditioned media or in presence of Ado. The 25th to 75th percentiles, the median and min-max of the values are represented; n = 12. *P < 0.05, **P < 0.01, Wilcoxon test. (e) Cumulative data showing the fold change in IFN-γ production by CD8+ T cells stimulated overnight with anti-CD3/anti-CD28 coated beads or PMA/Ionomycin in presence of Ado. The 25th to 75th percentiles, the median and min-max of the values are represented; n = 7. ***P < 0.001, one-way ANOVA test. (f) Cumulative data of the fold change in cytokine production (IL-2 and TNF-α) and CD107 expression after overnight stimulation with anti-CD3/anti-CD28 coated beads in presence of Ado in distinct memory CD8+ T-cell subsets (TCM, TEM, TEMRA). The 25th to 75th percentiles, the median and min-max of the values are represented; n = 12. *P < 0.05, ****P < 0.0001, one-way ANOVA test. Figure S2. Effects of Ado on CD8+ T cell functional avidity and evaluation of AdoR expression. (a) Cumulative data of the functional sensitivity (IC50 of IL-2 and TNF- α production) to Ad [file 40425_2019_719_MOESM1_ESM.zip › Supplementary Fig2 legend.docx]

**Supplementary Fig. 2** Effects of Ado on CD8^+^ T cell functional avidity and evaluation of AdoR expression. (**a**) Cumulative data of the functional sensitivity (IC_50_ of IL-2 and TNF- α production) to Ado measured in distinct CD8^+^ T cell memory subsets after overnight stimulation with anti-CD3/CD28 beads in presence of decreasing concentrations of Ado. The 25th to 75th percentiles, the median and min-max of the values are represented; *n* = 12. **P* < 0.05, *****P* < 0.0001, one-way ANOVA test. (**b**) Representative example of AdoR expression measured by RNA flow in total CD8 T cells. (**c**) Cumulative data of the AdoR expression in EBV transformed B cell line, THP-1, HL-60, PC-3, LNCAP, DU145, MCF-7 measured by qRT-PCR. (**d-e**) Correlation between AdoR expression measured by RNA flow and qRT-PCR in (**d**) cell lines and (**e**) primary human CD8 T cells. Spearman tests. (**f**) Represenative example of flow cytometry staining of total CD8 T cells by anti-A2AR and anti-A2BR antibodies. (**g**) Correlation between the expression of A2AR measured by antibody staining and RNA staining for flow cytometry in total CD8^+^ T cells. Spearman test. (**h**). Cumulative data of the expression of A2AR and A2BR in total CD8^+^ T cells measured by antibody staining for flow cytometry. The 25th to 75th percentiles, the median and min-max of the values are represented; *n* = 9.
