# Supplementary material for: Adenosine mediates functional and metabolic suppression of peripheral and tumor-infiltrating CD8+ T cells
Source: J Immunother Cancer. 2019 Oct 10;7:257. doi: 10.1186/s40425-019-0719-5 (PMC6788118; doi:10.1186/s40425-019-0719-5)
Supplement: Supplementary file 1 — Figure S1. Effects of Ado on CD8+ T cell cytokine production capacity. (a) Representative example of CD8+ T cell differentiation subsets identification by flow cytometry. (b) Representative example of cytokine production (i.e. IFN-γ, TNF-α, IL-2 and CD107) by CD8+ T cells stimulated overnight with anti-CD3/anti-CD28 coated beads or PMA/Ionomycin in presence or not of Ado. (c) Cumulative data showing the fold change in cytokine production (IL-2 and TNF-α) and CD107 expression by CD8+ T cells stimulated overnight with virus-specific peptides (n = 11) or anti-CD3/anti-CD28 coated beads (n = 12) in unconditioned media or in presence of Ado. The 25th to 75th percentiles, the median and min-max of the values are represented. ***P < 0.001, ****P < 0.0001, one-way ANOVA test. (d) Cumulative data showing the frequency of cytokine production (IL-2 and TNF-α) and CD107 expression by CD8+ T cells stimulated overnight with anti-CD3/anti-CD28 coated beads in unconditioned media or in presence of Ado. The 25th to 75th percentiles, the median and min-max of the values are represented; n = 12. *P < 0.05, **P < 0.01, Wilcoxon test. (e) Cumulative data showing the fold change in IFN-γ production by CD8+ T cells stimulated overnight with anti-CD3/anti-CD28 coated beads or PMA/Ionomycin in presence of Ado. The 25th to 75th percentiles, the median and min-max of the values are represented; n = 7. ***P < 0.001, one-way ANOVA test. (f) Cumulative data of the fold change in cytokine production (IL-2 and TNF-α) and CD107 expression after overnight stimulation with anti-CD3/anti-CD28 coated beads in presence of Ado in distinct memory CD8+ T-cell subsets (TCM, TEM, TEMRA). The 25th to 75th percentiles, the median and min-max of the values are represented; n = 12. *P < 0.05, ****P < 0.0001, one-way ANOVA test. Figure S2. Effects of Ado on CD8+ T cell functional avidity and evaluation of AdoR expression. (a) Cumulative data of the functional sensitivity (IC50 of IL-2 and TNF- α production) to Ad [file 40425_2019_719_MOESM1_ESM.zip › Supplementary Fig3 legend.docx]

**Supplementary Fig. 3** Functional consequences of AdoRs signaling. (**a**) Correlation between A2BR expression measured by RNA flow and the Ado-mediated fold change in IFN-γ production evaluated in total CD8^+^ T cells. Spearman test, *n* = 9. (**b**) Cumulative data showing the fold change in IFN-γ production by distinct memory CD8^+^ T cell subsets (T_CM_, T_EM_, T_EMRA_) stimulated with anti-CD3/anti-CD28 coated beads in presence of the A2AR selective agonist CGS 21680. The 25th to 75th percentiles, the median and min-max of the values are represented; *n* = 6. ***P* < 0.01, ****P* < 0.001, one-way ANOVA test. (**c**) Cumulative data showing the fold change in IFN-γ production by CD8^+^ T cells stimulated with anti-CD3/anti-CD28 coated beads in presence of Ado alone, or the A2AR selective antagonist (ZM 241385) alone, or the A2BR selective antagonist (PSB 1115) alone. The 25th to 75th percentiles, the median and min-max of the values are represented; *n* = 6. *****P* < 0.0001, one-way ANOVA test.
