# Supplementary material for: Adenosine mediates functional and metabolic suppression of peripheral and tumor-infiltrating CD8+ T cells
Source: J Immunother Cancer. 2019 Oct 10;7:257. doi: 10.1186/s40425-019-0719-5 (PMC6788118; doi:10.1186/s40425-019-0719-5)
Supplement: Supplementary file 1 — Figure S1. Effects of Ado on CD8+ T cell cytokine production capacity. (a) Representative example of CD8+ T cell differentiation subsets identification by flow cytometry. (b) Representative example of cytokine production (i.e. IFN-γ, TNF-α, IL-2 and CD107) by CD8+ T cells stimulated overnight with anti-CD3/anti-CD28 coated beads or PMA/Ionomycin in presence or not of Ado. (c) Cumulative data showing the fold change in cytokine production (IL-2 and TNF-α) and CD107 expression by CD8+ T cells stimulated overnight with virus-specific peptides (n = 11) or anti-CD3/anti-CD28 coated beads (n = 12) in unconditioned media or in presence of Ado. The 25th to 75th percentiles, the median and min-max of the values are represented. ***P < 0.001, ****P < 0.0001, one-way ANOVA test. (d) Cumulative data showing the frequency of cytokine production (IL-2 and TNF-α) and CD107 expression by CD8+ T cells stimulated overnight with anti-CD3/anti-CD28 coated beads in unconditioned media or in presence of Ado. The 25th to 75th percentiles, the median and min-max of the values are represented; n = 12. *P < 0.05, **P < 0.01, Wilcoxon test. (e) Cumulative data showing the fold change in IFN-γ production by CD8+ T cells stimulated overnight with anti-CD3/anti-CD28 coated beads or PMA/Ionomycin in presence of Ado. The 25th to 75th percentiles, the median and min-max of the values are represented; n = 7. ***P < 0.001, one-way ANOVA test. (f) Cumulative data of the fold change in cytokine production (IL-2 and TNF-α) and CD107 expression after overnight stimulation with anti-CD3/anti-CD28 coated beads in presence of Ado in distinct memory CD8+ T-cell subsets (TCM, TEM, TEMRA). The 25th to 75th percentiles, the median and min-max of the values are represented; n = 12. *P < 0.05, ****P < 0.0001, one-way ANOVA test. Figure S2. Effects of Ado on CD8+ T cell functional avidity and evaluation of AdoR expression. (a) Cumulative data of the functional sensitivity (IC50 of IL-2 and TNF- α production) to Ad [file 40425_2019_719_MOESM1_ESM.zip › Supplementary Fig5 legend.docx]

**Supplementary Fig. 5** Ado/A2AR impact p-S6 activation. The data represented in this figure were obtained analyzing CD8^+^ T cells stimulated for 3h by anti-CD3/anti-CD28 coated beads under the distinct described conditions. (**a**) Representative example of p-S6 expression detected by flow cytometry after treatment with Ado or the depicted combination of A2AR agonist (CGS 21680) and A2AR/A2BR antagonists (ZM 241385 and PSB 1115). (**b**) Representative western blot analysis of p-S6 and S6 after treatment with Ado alone or combined with the A2AR antagonist (ZM 241385) or the PKA inhibitor (KT570), Rapamycin, the AKT1/2 inhibitor (MK2206). α-Tubulin was detected as a loading control. *n* = 3. (**c**) Representative example of p-Akt^Ser473^ expression detected by flow cytometry in resting condition or after treatment with Ado. (**d**) Representative western blot analysis of p-Akt^Ser473^ and Akt^Ser473^ after treatment with Ado alone or combined with the A2AR antagonist (ZM 241385) or the PKA inhibitor (KT570), Rapamycin, the AKT1/2 inhibitor (MK2206). α-Tubulin was detected as a loading control. *n* = 3. (**e**) Cumulative data showing the fold change in p-Akt^Ser473^ expression after treatment with the indicated combinations of Ado, A2AR agonist (CGS 21680), A2AR/A2BR antagonists (ZM 241385 and PSB 1115, respectively) or the AKT_1/2_ inhibitor (MK2206). The 25th to 75th percentiles, the median and min-max of the values are represented; *n* = 7. (**f**) Cumulative data showing the fold change in p-S6 expression by memory CD8^+^ T cell subsets (T_CM_, T_EM_, T_EMRA_) in presence of Ado or the A2AR selective agonist CGS 21680. The 25th to 75th percentiles, the median and min-max of the values are represented; *n* = 14 and *n* = 12 from left to right. ***P* < 0.01, ****P* < 0.001, *****P* < 0.0001, one-way ANOVA test.
