# Supplementary material for: Adenosine mediates functional and metabolic suppression of peripheral and tumor-infiltrating CD8+ T cells
Source: J Immunother Cancer. 2019 Oct 10;7:257. doi: 10.1186/s40425-019-0719-5 (PMC6788118; doi:10.1186/s40425-019-0719-5)
Supplement: Supplementary file 1 — Figure S1. Effects of Ado on CD8+ T cell cytokine production capacity. (a) Representative example of CD8+ T cell differentiation subsets identification by flow cytometry. (b) Representative example of cytokine production (i.e. IFN-γ, TNF-α, IL-2 and CD107) by CD8+ T cells stimulated overnight with anti-CD3/anti-CD28 coated beads or PMA/Ionomycin in presence or not of Ado. (c) Cumulative data showing the fold change in cytokine production (IL-2 and TNF-α) and CD107 expression by CD8+ T cells stimulated overnight with virus-specific peptides (n = 11) or anti-CD3/anti-CD28 coated beads (n = 12) in unconditioned media or in presence of Ado. The 25th to 75th percentiles, the median and min-max of the values are represented. ***P < 0.001, ****P < 0.0001, one-way ANOVA test. (d) Cumulative data showing the frequency of cytokine production (IL-2 and TNF-α) and CD107 expression by CD8+ T cells stimulated overnight with anti-CD3/anti-CD28 coated beads in unconditioned media or in presence of Ado. The 25th to 75th percentiles, the median and min-max of the values are represented; n = 12. *P < 0.05, **P < 0.01, Wilcoxon test. (e) Cumulative data showing the fold change in IFN-γ production by CD8+ T cells stimulated overnight with anti-CD3/anti-CD28 coated beads or PMA/Ionomycin in presence of Ado. The 25th to 75th percentiles, the median and min-max of the values are represented; n = 7. ***P < 0.001, one-way ANOVA test. (f) Cumulative data of the fold change in cytokine production (IL-2 and TNF-α) and CD107 expression after overnight stimulation with anti-CD3/anti-CD28 coated beads in presence of Ado in distinct memory CD8+ T-cell subsets (TCM, TEM, TEMRA). The 25th to 75th percentiles, the median and min-max of the values are represented; n = 12. *P < 0.05, ****P < 0.0001, one-way ANOVA test. Figure S2. Effects of Ado on CD8+ T cell functional avidity and evaluation of AdoR expression. (a) Cumulative data of the functional sensitivity (IC50 of IL-2 and TNF- α production) to Ad [file 40425_2019_719_MOESM1_ESM.zip › Supplementary Fig7 legend.docx]

**Supplementary Fig. 7** Composition, functionality and AdoR expression in TILs. (**a**) Proportion of CD4^+^ and CD8^+^ T cells in REP expanded TILs derived from normal/inflamed or tumor prostate tissue and metastasis of colon cancer or of melanoma. (**b**) Cumulative data of the frequency of cytokine-producing or CD107^+^ CD8^+^ TILs. Each dot represents a patient, lines indicate Mean±SEM; *n* = 1. (**c**) Cumulative data of the fold change in IL-2, TNF-α production and CD107 expression by TILs stimulated overnight with autologous tumor cells in presence of Ado or ZM 241385+Ado or PSB 1115+Ado. Each dot represents a patient, lines indicate Mean±SEM; *n* = 14. ***P* < 0.01, ****P* < 0.001, *****P* < 0.0001, *****P* < 0.0001, one-way ANOVA test. (**d**) Cumulative data of the expression of AdoR in peripheral T cells from healthy donors and in TILs derived from the depicted tissue types. The 25th to 75th percentiles, the median and min-max of the values are represented by boxes. Each dot represents a patient. The positivity threshold is determined as 2 log higher expression than values given by water. (**e**) Cumulative data of the expression of AdoR in peripheral CD8^+^ T cells from healthy donors at day 0 or after 3 and 10 days stimulation with anti-CD3/anti-CD28 coated beads. Each dot represents the mean of 8 healthy donors analyzed. **P* < 0.05, one-way ANOVA test. (**f**) Correlation between the A2AR and HIF-1α expression measured by qRT-PCR in TILs. Spearman test, *n* = 13.
