# Supplementary material for: Adenosine mediates functional and metabolic suppression of peripheral and tumor-infiltrating CD8+ T cells
Source: J Immunother Cancer. 2019 Oct 10;7:257. doi: 10.1186/s40425-019-0719-5 (PMC6788118; doi:10.1186/s40425-019-0719-5)
Supplement: Supplementary file 1 — Figure S1. Effects of Ado on CD8+ T cell cytokine production capacity. (a) Representative example of CD8+ T cell differentiation subsets identification by flow cytometry. (b) Representative example of cytokine production (i.e. IFN-γ, TNF-α, IL-2 and CD107) by CD8+ T cells stimulated overnight with anti-CD3/anti-CD28 coated beads or PMA/Ionomycin in presence or not of Ado. (c) Cumulative data showing the fold change in cytokine production (IL-2 and TNF-α) and CD107 expression by CD8+ T cells stimulated overnight with virus-specific peptides (n = 11) or anti-CD3/anti-CD28 coated beads (n = 12) in unconditioned media or in presence of Ado. The 25th to 75th percentiles, the median and min-max of the values are represented. ***P < 0.001, ****P < 0.0001, one-way ANOVA test. (d) Cumulative data showing the frequency of cytokine production (IL-2 and TNF-α) and CD107 expression by CD8+ T cells stimulated overnight with anti-CD3/anti-CD28 coated beads in unconditioned media or in presence of Ado. The 25th to 75th percentiles, the median and min-max of the values are represented; n = 12. *P < 0.05, **P < 0.01, Wilcoxon test. (e) Cumulative data showing the fold change in IFN-γ production by CD8+ T cells stimulated overnight with anti-CD3/anti-CD28 coated beads or PMA/Ionomycin in presence of Ado. The 25th to 75th percentiles, the median and min-max of the values are represented; n = 7. ***P < 0.001, one-way ANOVA test. (f) Cumulative data of the fold change in cytokine production (IL-2 and TNF-α) and CD107 expression after overnight stimulation with anti-CD3/anti-CD28 coated beads in presence of Ado in distinct memory CD8+ T-cell subsets (TCM, TEM, TEMRA). The 25th to 75th percentiles, the median and min-max of the values are represented; n = 12. *P < 0.05, ****P < 0.0001, one-way ANOVA test. Figure S2. Effects of Ado on CD8+ T cell functional avidity and evaluation of AdoR expression. (a) Cumulative data of the functional sensitivity (IC50 of IL-2 and TNF- α production) to Ad [file 40425_2019_719_MOESM1_ESM.zip › Supplementary Fig8 legend.docx]

**Supplementary Fig. 8** AdoR expression and Ado immunosuppression in TILs/autologous tumor cells setting. (**a**) Cumulative data of the expression of AdoR in peripheral melanoma-derived TILs and tumor cells. The 25th to 75th percentiles, the median and min-max of the values are represented by boxes; *n* = 6. (**b**) Representative example of cytokine production in melanoma-derived TILs after overnight stimulation with autologous tumor cells in untreated culture condition, in presence of Ado or ZM 241385+Ado. (**c**) Representative example of CD71 and CD98 expression by TILs stimulated for overnight by autologous tumor cells in resting condition, in presence of Ado or ZM 241385+Ado. (**d**) Each graph represents cytotoxicity curves for one patient quantified in resting condition, in presence of Ado or ZM 241385+Ado. Data are represented as normalized to the cytotoxicity measured in absence of Ado at the effector:target (E:T) ratio 100:1. Cytotoxicity was measured co-incubating TILs for 4h with P815 cell line loaded with anti-CD3 (redirected killing; bottom graphs); n = 3, measures were performed in triplicates. (**e**) Representative example of p-CREB expression by TILs in untreated culture condition or in presence of Ado or ZM 241385+Ado. (**f**) Representative example of p-S6 and CD107 expression by TILs stimulated for 3h by autologous tumor cells in untreated culture condition or in presence of Ado or ZM 241385+Ado.
