# Supplementary figures and images for: Adenosine mediates functional and metabolic suppression of peripheral and tumor-infiltrating CD8+ T cells
Source: J Immunother Cancer. 2019 Oct 10;7:257. doi: 10.1186/s40425-019-0719-5 (PMC6788118; doi:10.1186/s40425-019-0719-5)

**Supplementary Fig. 1**

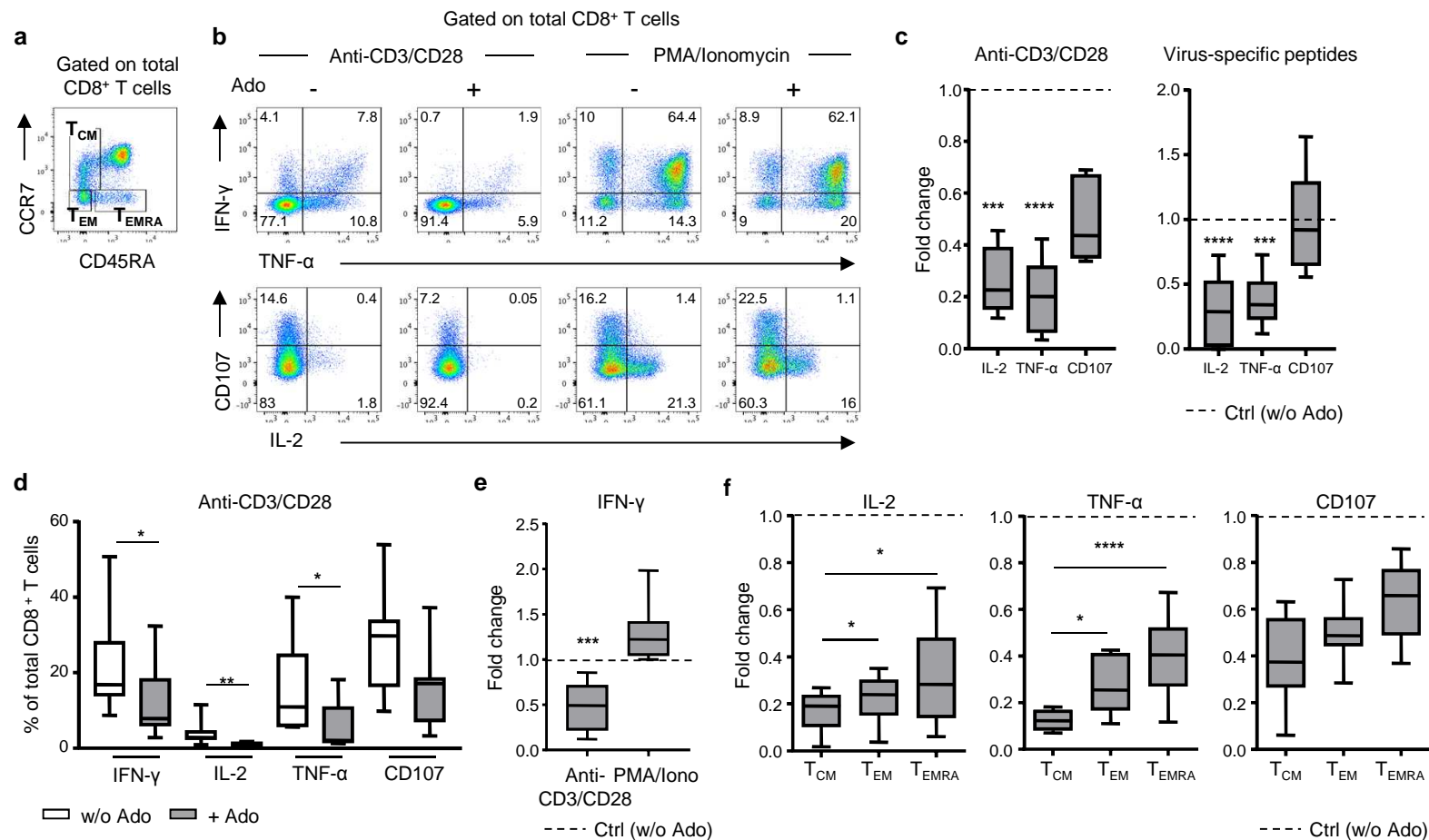

Supplement: Supplementary file 1 — Figure S1. Effects of Ado on CD8+ T cell cytokine production capacity. (a) Representative example of CD8+ T cell differentiation subsets identification by flow cytometry. (b) Representative example of cytokine production (i.e. IFN-γ, TNF-α, IL-2 and CD107) by CD8+ T cells stimulated overnight with anti-CD3/anti-CD28 coated beads or PMA/Ionomycin in presence or not of Ado. (c) Cumulative data showing the fold change in cytokine production (IL-2 and TNF-α) and CD107 expression by CD8+ T cells stimulated overnight with virus-specific peptides (n = 11) or anti-CD3/anti-CD28 coated beads (n = 12) in unconditioned media or in presence of Ado. The 25th to 75th percentiles, the median and min-max of the values are represented. ***P < 0.001, ****P < 0.0001, one-way ANOVA test. (d) Cumulative data showing the frequency of cytokine production (IL-2 and TNF-α) and CD107 expression by CD8+ T cells stimulated overnight with anti-CD3/anti-CD28 coated beads in unconditioned media or in presence of Ado. The 25th to 75th percentiles, the median and min-max of the values are represented; n = 12. *P < 0.05, **P < 0.01, Wilcoxon test. (e) Cumulative data showing the fold change in IFN-γ production by CD8+ T cells stimulated overnight with anti-CD3/anti-CD28 coated beads or PMA/Ionomycin in presence of Ado. The 25th to 75th percentiles, the median and min-max of the values are represented; n = 7. ***P < 0.001, one-way ANOVA test. (f) Cumulative data of the fold change in cytokine production (IL-2 and TNF-α) and CD107 expression after overnight stimulation with anti-CD3/anti-CD28 coated beads in presence of Ado in distinct memory CD8+ T-cell subsets (TCM, TEM, TEMRA). The 25th to 75th percentiles, the median and min-max of the values are represented; n = 12. *P < 0.05, ****P < 0.0001, one-way ANOVA test. Figure S2. Effects of Ado on CD8+ T cell functional avidity and evaluation of AdoR expression. (a) Cumulative data of the functional sensitivity (IC50 of IL-2 and TNF- α production) to Ad [file 40425_2019_719_MOESM1_ESM.zip › Mastelic, Navarro et al_Suppl figure1.pdf]

**Supplementary Fig. 2**

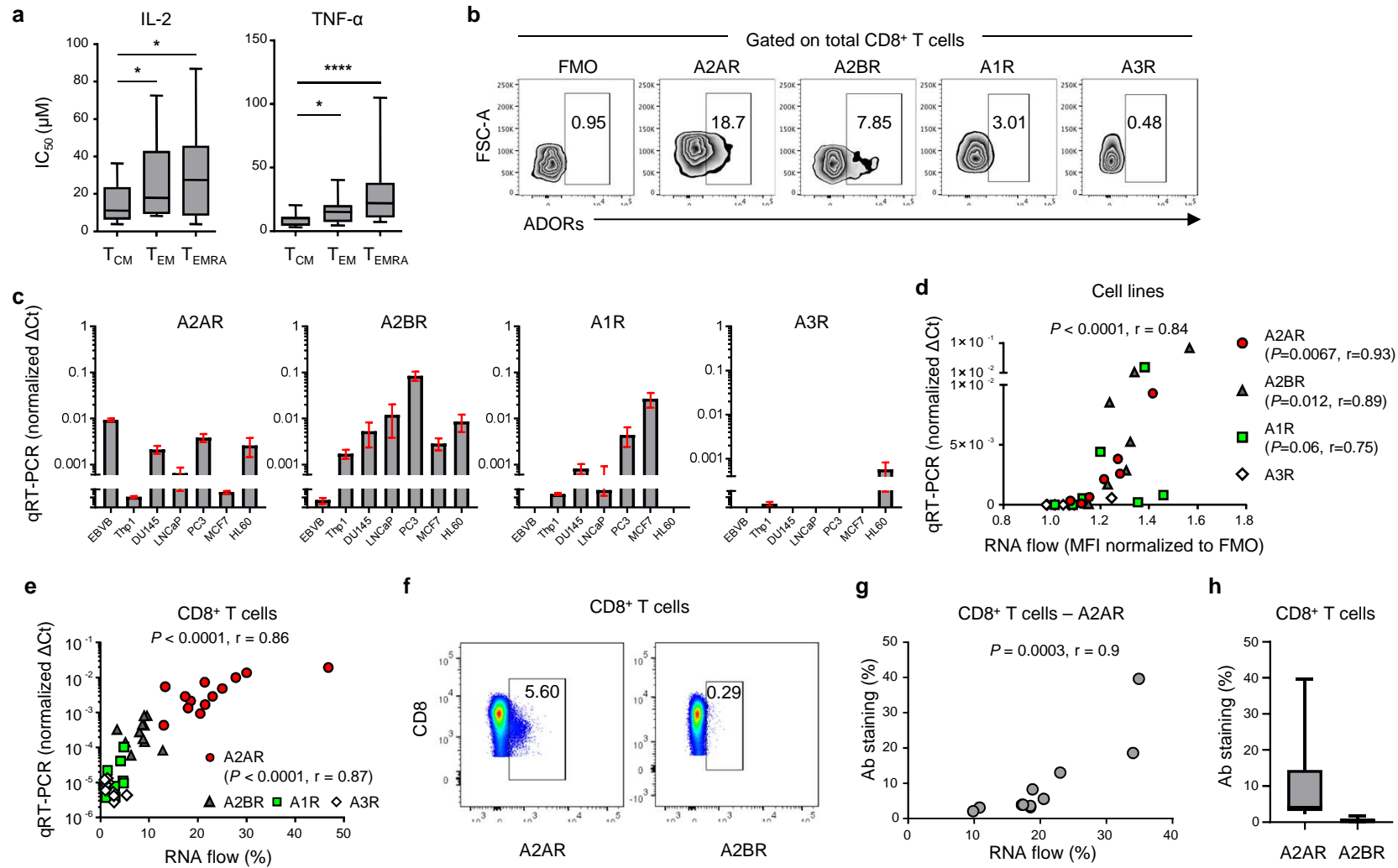

Supplement: Supplementary file 1 — Figure S1. Effects of Ado on CD8+ T cell cytokine production capacity. (a) Representative example of CD8+ T cell differentiation subsets identification by flow cytometry. (b) Representative example of cytokine production (i.e. IFN-γ, TNF-α, IL-2 and CD107) by CD8+ T cells stimulated overnight with anti-CD3/anti-CD28 coated beads or PMA/Ionomycin in presence or not of Ado. (c) Cumulative data showing the fold change in cytokine production (IL-2 and TNF-α) and CD107 expression by CD8+ T cells stimulated overnight with virus-specific peptides (n = 11) or anti-CD3/anti-CD28 coated beads (n = 12) in unconditioned media or in presence of Ado. The 25th to 75th percentiles, the median and min-max of the values are represented. ***P < 0.001, ****P < 0.0001, one-way ANOVA test. (d) Cumulative data showing the frequency of cytokine production (IL-2 and TNF-α) and CD107 expression by CD8+ T cells stimulated overnight with anti-CD3/anti-CD28 coated beads in unconditioned media or in presence of Ado. The 25th to 75th percentiles, the median and min-max of the values are represented; n = 12. *P < 0.05, **P < 0.01, Wilcoxon test. (e) Cumulative data showing the fold change in IFN-γ production by CD8+ T cells stimulated overnight with anti-CD3/anti-CD28 coated beads or PMA/Ionomycin in presence of Ado. The 25th to 75th percentiles, the median and min-max of the values are represented; n = 7. ***P < 0.001, one-way ANOVA test. (f) Cumulative data of the fold change in cytokine production (IL-2 and TNF-α) and CD107 expression after overnight stimulation with anti-CD3/anti-CD28 coated beads in presence of Ado in distinct memory CD8+ T-cell subsets (TCM, TEM, TEMRA). The 25th to 75th percentiles, the median and min-max of the values are represented; n = 12. *P < 0.05, ****P < 0.0001, one-way ANOVA test. Figure S2. Effects of Ado on CD8+ T cell functional avidity and evaluation of AdoR expression. (a) Cumulative data of the functional sensitivity (IC50 of IL-2 and TNF- α production) to Ad [file 40425_2019_719_MOESM1_ESM.zip › Mastelic, Navarro et al_Suppl figure2.pdf]

**Supplementary Fig. 3**

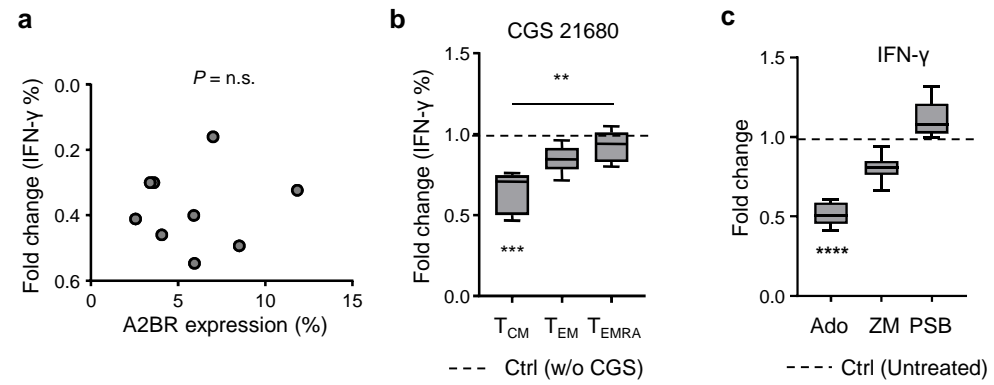

Supplement: Supplementary file 1 — Figure S1. Effects of Ado on CD8+ T cell cytokine production capacity. (a) Representative example of CD8+ T cell differentiation subsets identification by flow cytometry. (b) Representative example of cytokine production (i.e. IFN-γ, TNF-α, IL-2 and CD107) by CD8+ T cells stimulated overnight with anti-CD3/anti-CD28 coated beads or PMA/Ionomycin in presence or not of Ado. (c) Cumulative data showing the fold change in cytokine production (IL-2 and TNF-α) and CD107 expression by CD8+ T cells stimulated overnight with virus-specific peptides (n = 11) or anti-CD3/anti-CD28 coated beads (n = 12) in unconditioned media or in presence of Ado. The 25th to 75th percentiles, the median and min-max of the values are represented. ***P < 0.001, ****P < 0.0001, one-way ANOVA test. (d) Cumulative data showing the frequency of cytokine production (IL-2 and TNF-α) and CD107 expression by CD8+ T cells stimulated overnight with anti-CD3/anti-CD28 coated beads in unconditioned media or in presence of Ado. The 25th to 75th percentiles, the median and min-max of the values are represented; n = 12. *P < 0.05, **P < 0.01, Wilcoxon test. (e) Cumulative data showing the fold change in IFN-γ production by CD8+ T cells stimulated overnight with anti-CD3/anti-CD28 coated beads or PMA/Ionomycin in presence of Ado. The 25th to 75th percentiles, the median and min-max of the values are represented; n = 7. ***P < 0.001, one-way ANOVA test. (f) Cumulative data of the fold change in cytokine production (IL-2 and TNF-α) and CD107 expression after overnight stimulation with anti-CD3/anti-CD28 coated beads in presence of Ado in distinct memory CD8+ T-cell subsets (TCM, TEM, TEMRA). The 25th to 75th percentiles, the median and min-max of the values are represented; n = 12. *P < 0.05, ****P < 0.0001, one-way ANOVA test. Figure S2. Effects of Ado on CD8+ T cell functional avidity and evaluation of AdoR expression. (a) Cumulative data of the functional sensitivity (IC50 of IL-2 and TNF- α production) to Ad [file 40425_2019_719_MOESM1_ESM.zip › Mastelic, Navarro et al_Suppl figure3.pdf]

Supplementary Fig. 4

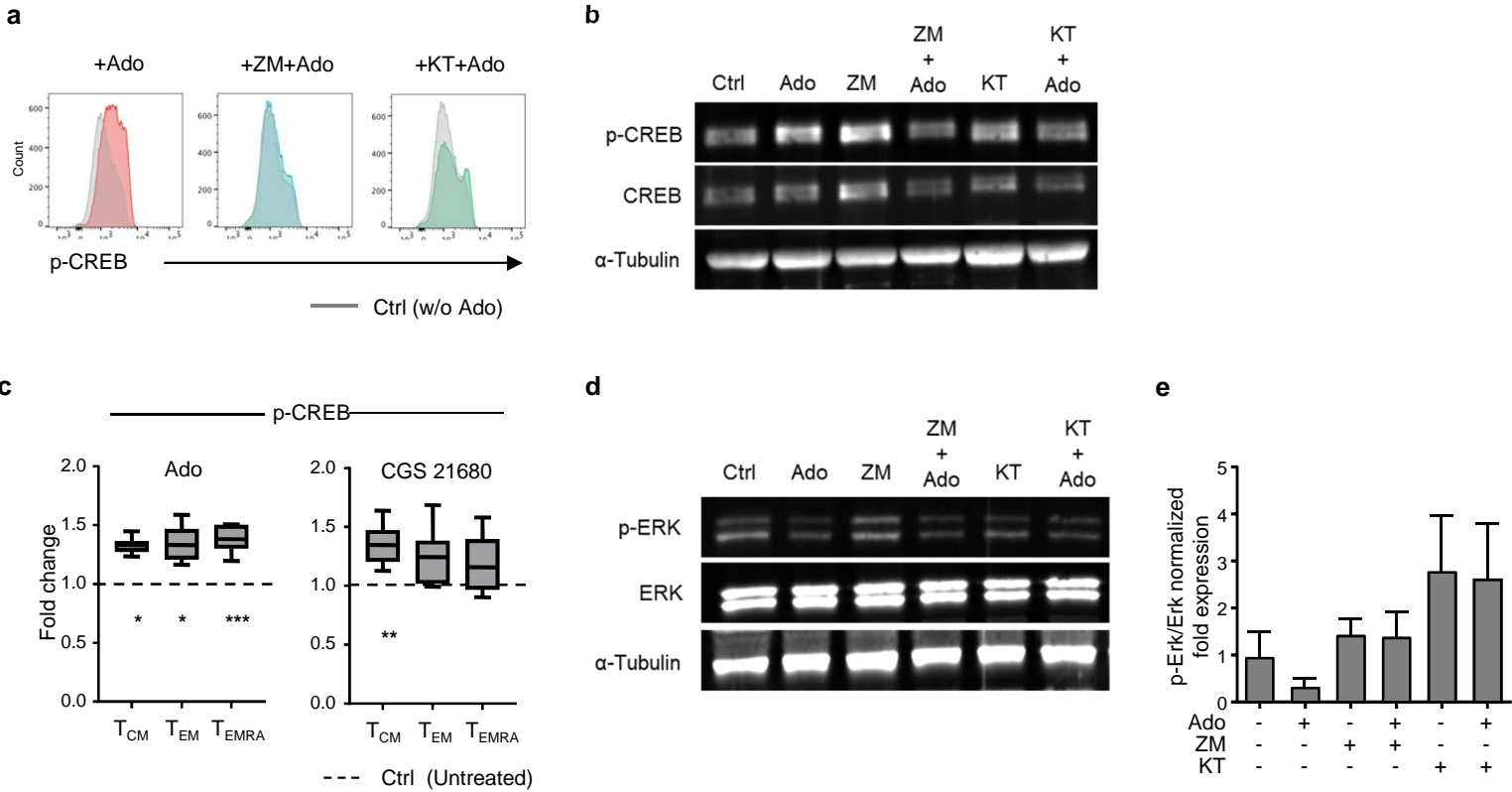

Supplement: Supplementary file 1 — Figure S1. Effects of Ado on CD8+ T cell cytokine production capacity. (a) Representative example of CD8+ T cell differentiation subsets identification by flow cytometry. (b) Representative example of cytokine production (i.e. IFN-γ, TNF-α, IL-2 and CD107) by CD8+ T cells stimulated overnight with anti-CD3/anti-CD28 coated beads or PMA/Ionomycin in presence or not of Ado. (c) Cumulative data showing the fold change in cytokine production (IL-2 and TNF-α) and CD107 expression by CD8+ T cells stimulated overnight with virus-specific peptides (n = 11) or anti-CD3/anti-CD28 coated beads (n = 12) in unconditioned media or in presence of Ado. The 25th to 75th percentiles, the median and min-max of the values are represented. ***P < 0.001, ****P < 0.0001, one-way ANOVA test. (d) Cumulative data showing the frequency of cytokine production (IL-2 and TNF-α) and CD107 expression by CD8+ T cells stimulated overnight with anti-CD3/anti-CD28 coated beads in unconditioned media or in presence of Ado. The 25th to 75th percentiles, the median and min-max of the values are represented; n = 12. *P < 0.05, **P < 0.01, Wilcoxon test. (e) Cumulative data showing the fold change in IFN-γ production by CD8+ T cells stimulated overnight with anti-CD3/anti-CD28 coated beads or PMA/Ionomycin in presence of Ado. The 25th to 75th percentiles, the median and min-max of the values are represented; n = 7. ***P < 0.001, one-way ANOVA test. (f) Cumulative data of the fold change in cytokine production (IL-2 and TNF-α) and CD107 expression after overnight stimulation with anti-CD3/anti-CD28 coated beads in presence of Ado in distinct memory CD8+ T-cell subsets (TCM, TEM, TEMRA). The 25th to 75th percentiles, the median and min-max of the values are represented; n = 12. *P < 0.05, ****P < 0.0001, one-way ANOVA test. Figure S2. Effects of Ado on CD8+ T cell functional avidity and evaluation of AdoR expression. (a) Cumulative data of the functional sensitivity (IC50 of IL-2 and TNF- α production) to Ad [file 40425_2019_719_MOESM1_ESM.zip › Mastelic, Navarro et al_Suppl figure4.pdf]

**Supplementary Fig. 5**

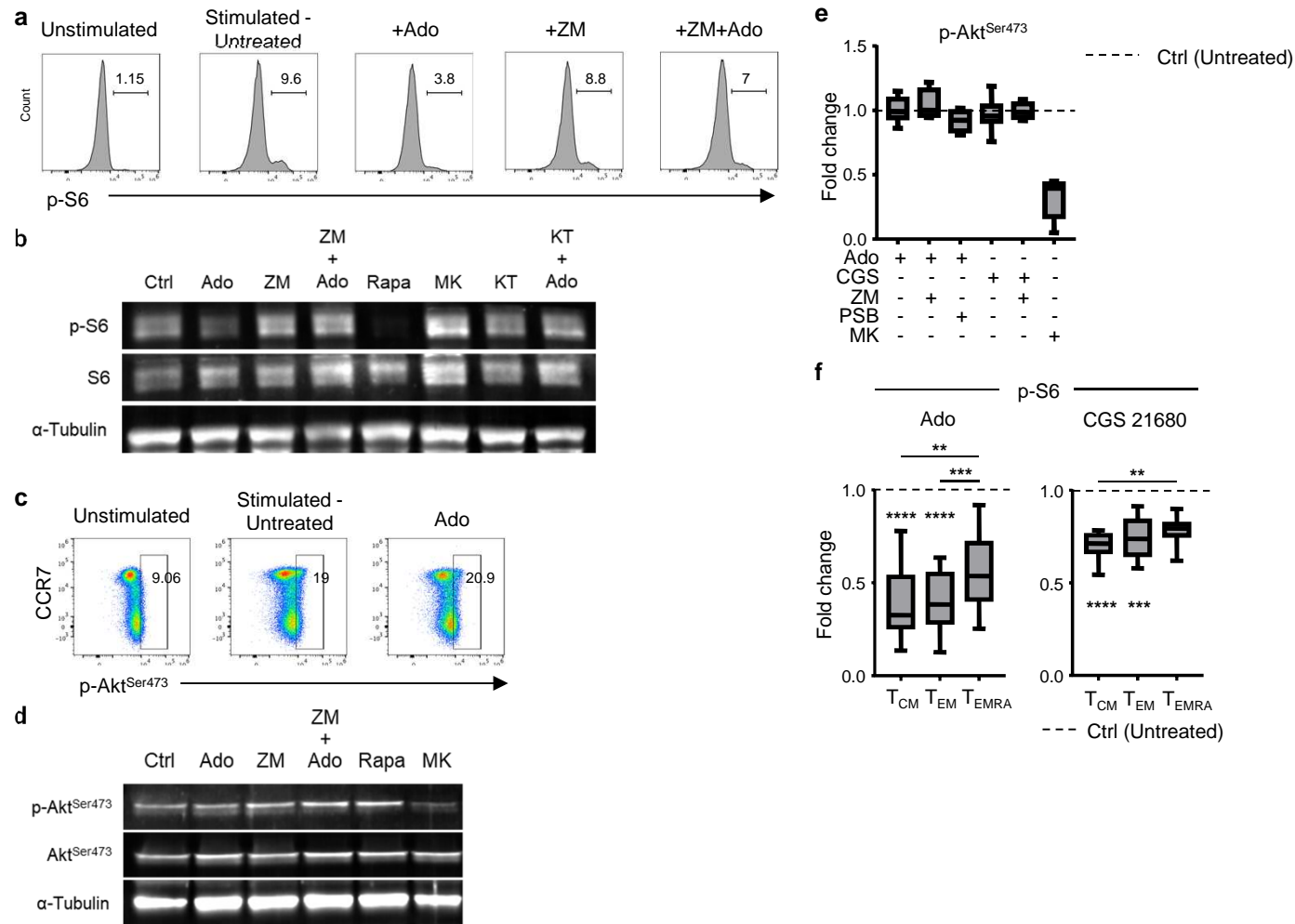

Supplement: Supplementary file 1 — Figure S1. Effects of Ado on CD8+ T cell cytokine production capacity. (a) Representative example of CD8+ T cell differentiation subsets identification by flow cytometry. (b) Representative example of cytokine production (i.e. IFN-γ, TNF-α, IL-2 and CD107) by CD8+ T cells stimulated overnight with anti-CD3/anti-CD28 coated beads or PMA/Ionomycin in presence or not of Ado. (c) Cumulative data showing the fold change in cytokine production (IL-2 and TNF-α) and CD107 expression by CD8+ T cells stimulated overnight with virus-specific peptides (n = 11) or anti-CD3/anti-CD28 coated beads (n = 12) in unconditioned media or in presence of Ado. The 25th to 75th percentiles, the median and min-max of the values are represented. ***P < 0.001, ****P < 0.0001, one-way ANOVA test. (d) Cumulative data showing the frequency of cytokine production (IL-2 and TNF-α) and CD107 expression by CD8+ T cells stimulated overnight with anti-CD3/anti-CD28 coated beads in unconditioned media or in presence of Ado. The 25th to 75th percentiles, the median and min-max of the values are represented; n = 12. *P < 0.05, **P < 0.01, Wilcoxon test. (e) Cumulative data showing the fold change in IFN-γ production by CD8+ T cells stimulated overnight with anti-CD3/anti-CD28 coated beads or PMA/Ionomycin in presence of Ado. The 25th to 75th percentiles, the median and min-max of the values are represented; n = 7. ***P < 0.001, one-way ANOVA test. (f) Cumulative data of the fold change in cytokine production (IL-2 and TNF-α) and CD107 expression after overnight stimulation with anti-CD3/anti-CD28 coated beads in presence of Ado in distinct memory CD8+ T-cell subsets (TCM, TEM, TEMRA). The 25th to 75th percentiles, the median and min-max of the values are represented; n = 12. *P < 0.05, ****P < 0.0001, one-way ANOVA test. Figure S2. Effects of Ado on CD8+ T cell functional avidity and evaluation of AdoR expression. (a) Cumulative data of the functional sensitivity (IC50 of IL-2 and TNF- α production) to Ad [file 40425_2019_719_MOESM1_ESM.zip › Mastelic, Navarro et al_Suppl figure5.pdf]

## Supplementary Fig. 6

**a**

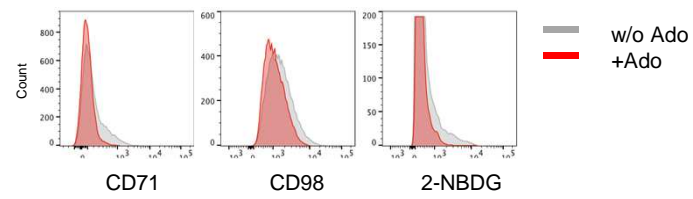

**b**

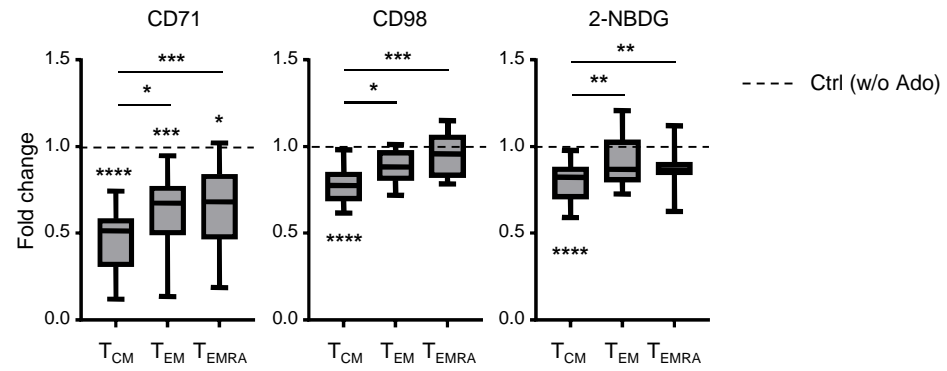

Supplement: Supplementary file 1 — Figure S1. Effects of Ado on CD8+ T cell cytokine production capacity. (a) Representative example of CD8+ T cell differentiation subsets identification by flow cytometry. (b) Representative example of cytokine production (i.e. IFN-γ, TNF-α, IL-2 and CD107) by CD8+ T cells stimulated overnight with anti-CD3/anti-CD28 coated beads or PMA/Ionomycin in presence or not of Ado. (c) Cumulative data showing the fold change in cytokine production (IL-2 and TNF-α) and CD107 expression by CD8+ T cells stimulated overnight with virus-specific peptides (n = 11) or anti-CD3/anti-CD28 coated beads (n = 12) in unconditioned media or in presence of Ado. The 25th to 75th percentiles, the median and min-max of the values are represented. ***P < 0.001, ****P < 0.0001, one-way ANOVA test. (d) Cumulative data showing the frequency of cytokine production (IL-2 and TNF-α) and CD107 expression by CD8+ T cells stimulated overnight with anti-CD3/anti-CD28 coated beads in unconditioned media or in presence of Ado. The 25th to 75th percentiles, the median and min-max of the values are represented; n = 12. *P < 0.05, **P < 0.01, Wilcoxon test. (e) Cumulative data showing the fold change in IFN-γ production by CD8+ T cells stimulated overnight with anti-CD3/anti-CD28 coated beads or PMA/Ionomycin in presence of Ado. The 25th to 75th percentiles, the median and min-max of the values are represented; n = 7. ***P < 0.001, one-way ANOVA test. (f) Cumulative data of the fold change in cytokine production (IL-2 and TNF-α) and CD107 expression after overnight stimulation with anti-CD3/anti-CD28 coated beads in presence of Ado in distinct memory CD8+ T-cell subsets (TCM, TEM, TEMRA). The 25th to 75th percentiles, the median and min-max of the values are represented; n = 12. *P < 0.05, ****P < 0.0001, one-way ANOVA test. Figure S2. Effects of Ado on CD8+ T cell functional avidity and evaluation of AdoR expression. (a) Cumulative data of the functional sensitivity (IC50 of IL-2 and TNF- α production) to Ad [file 40425_2019_719_MOESM1_ESM.zip › Mastelic, Navarro et al_Suppl figure6.pdf]

## Supplementary figure 7

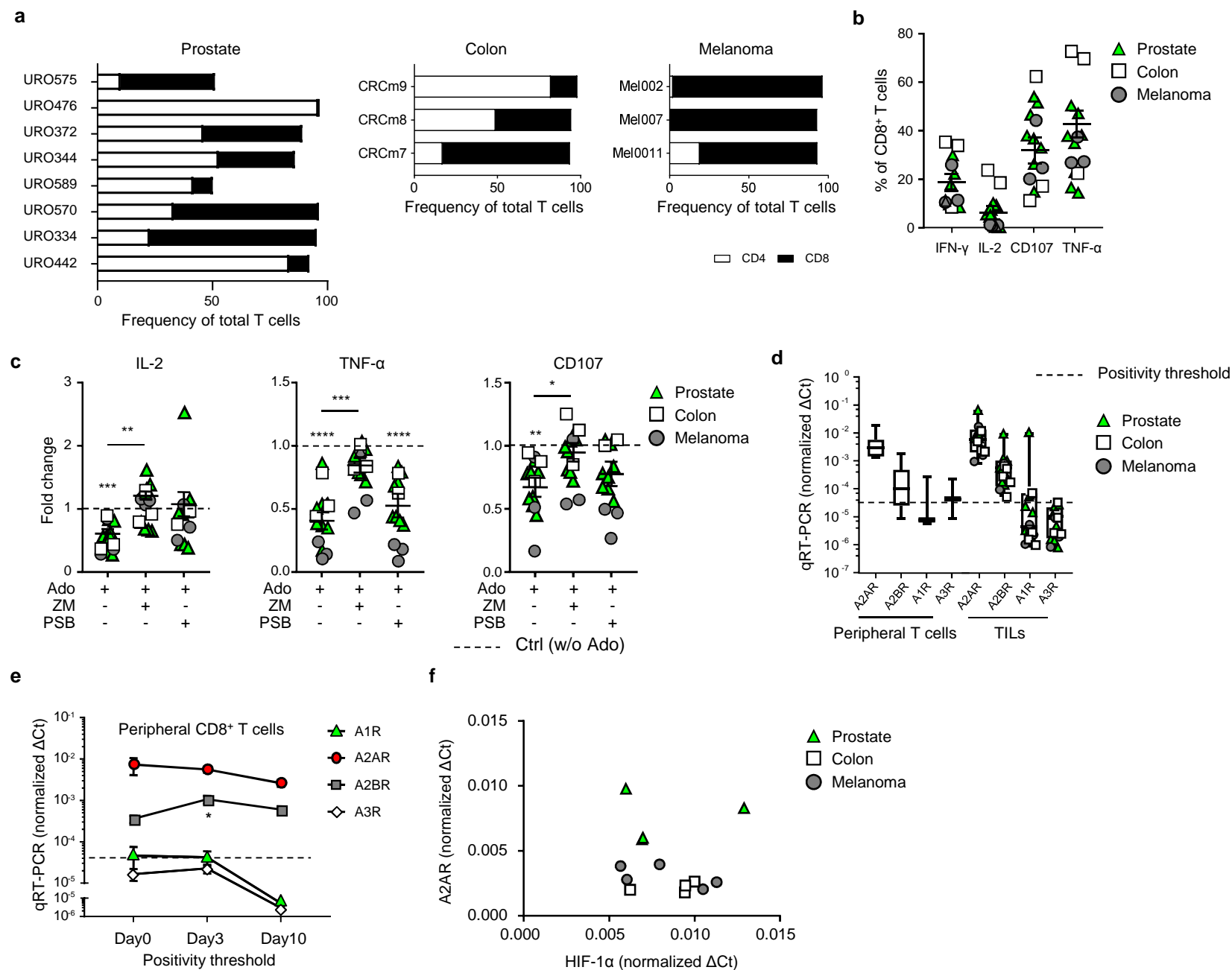

Supplement: Supplementary file 1 — Figure S1. Effects of Ado on CD8+ T cell cytokine production capacity. (a) Representative example of CD8+ T cell differentiation subsets identification by flow cytometry. (b) Representative example of cytokine production (i.e. IFN-γ, TNF-α, IL-2 and CD107) by CD8+ T cells stimulated overnight with anti-CD3/anti-CD28 coated beads or PMA/Ionomycin in presence or not of Ado. (c) Cumulative data showing the fold change in cytokine production (IL-2 and TNF-α) and CD107 expression by CD8+ T cells stimulated overnight with virus-specific peptides (n = 11) or anti-CD3/anti-CD28 coated beads (n = 12) in unconditioned media or in presence of Ado. The 25th to 75th percentiles, the median and min-max of the values are represented. ***P < 0.001, ****P < 0.0001, one-way ANOVA test. (d) Cumulative data showing the frequency of cytokine production (IL-2 and TNF-α) and CD107 expression by CD8+ T cells stimulated overnight with anti-CD3/anti-CD28 coated beads in unconditioned media or in presence of Ado. The 25th to 75th percentiles, the median and min-max of the values are represented; n = 12. *P < 0.05, **P < 0.01, Wilcoxon test. (e) Cumulative data showing the fold change in IFN-γ production by CD8+ T cells stimulated overnight with anti-CD3/anti-CD28 coated beads or PMA/Ionomycin in presence of Ado. The 25th to 75th percentiles, the median and min-max of the values are represented; n = 7. ***P < 0.001, one-way ANOVA test. (f) Cumulative data of the fold change in cytokine production (IL-2 and TNF-α) and CD107 expression after overnight stimulation with anti-CD3/anti-CD28 coated beads in presence of Ado in distinct memory CD8+ T-cell subsets (TCM, TEM, TEMRA). The 25th to 75th percentiles, the median and min-max of the values are represented; n = 12. *P < 0.05, ****P < 0.0001, one-way ANOVA test. Figure S2. Effects of Ado on CD8+ T cell functional avidity and evaluation of AdoR expression. (a) Cumulative data of the functional sensitivity (IC50 of IL-2 and TNF- α production) to Ad [file 40425_2019_719_MOESM1_ESM.zip › Mastelic, Navarro et al_Suppl figure7.pdf]

## Supplementary figure 8

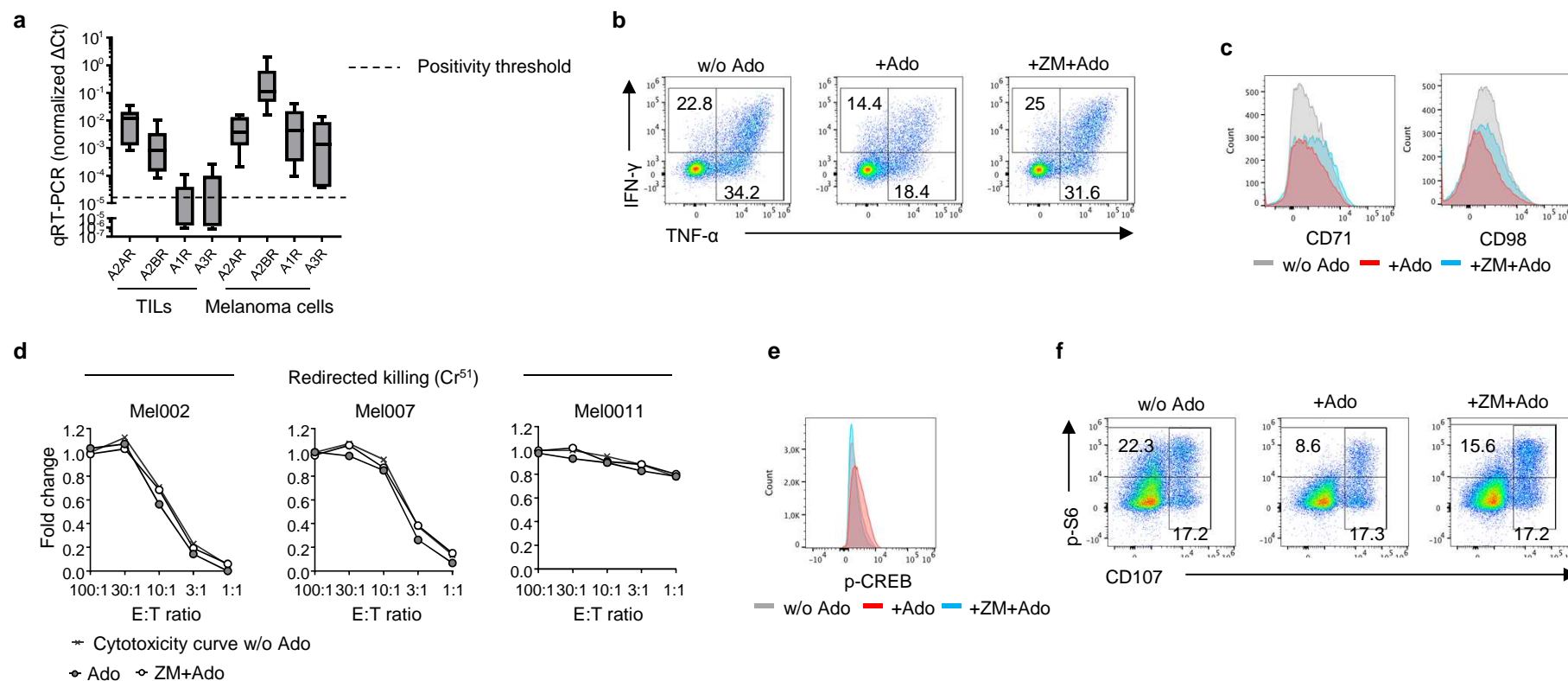

Supplement: Supplementary file 1 — Figure S1. Effects of Ado on CD8+ T cell cytokine production capacity. (a) Representative example of CD8+ T cell differentiation subsets identification by flow cytometry. (b) Representative example of cytokine production (i.e. IFN-γ, TNF-α, IL-2 and CD107) by CD8+ T cells stimulated overnight with anti-CD3/anti-CD28 coated beads or PMA/Ionomycin in presence or not of Ado. (c) Cumulative data showing the fold change in cytokine production (IL-2 and TNF-α) and CD107 expression by CD8+ T cells stimulated overnight with virus-specific peptides (n = 11) or anti-CD3/anti-CD28 coated beads (n = 12) in unconditioned media or in presence of Ado. The 25th to 75th percentiles, the median and min-max of the values are represented. ***P < 0.001, ****P < 0.0001, one-way ANOVA test. (d) Cumulative data showing the frequency of cytokine production (IL-2 and TNF-α) and CD107 expression by CD8+ T cells stimulated overnight with anti-CD3/anti-CD28 coated beads in unconditioned media or in presence of Ado. The 25th to 75th percentiles, the median and min-max of the values are represented; n = 12. *P < 0.05, **P < 0.01, Wilcoxon test. (e) Cumulative data showing the fold change in IFN-γ production by CD8+ T cells stimulated overnight with anti-CD3/anti-CD28 coated beads or PMA/Ionomycin in presence of Ado. The 25th to 75th percentiles, the median and min-max of the values are represented; n = 7. ***P < 0.001, one-way ANOVA test. (f) Cumulative data of the fold change in cytokine production (IL-2 and TNF-α) and CD107 expression after overnight stimulation with anti-CD3/anti-CD28 coated beads in presence of Ado in distinct memory CD8+ T-cell subsets (TCM, TEM, TEMRA). The 25th to 75th percentiles, the median and min-max of the values are represented; n = 12. *P < 0.05, ****P < 0.0001, one-way ANOVA test. Figure S2. Effects of Ado on CD8+ T cell functional avidity and evaluation of AdoR expression. (a) Cumulative data of the functional sensitivity (IC50 of IL-2 and TNF- α production) to Ad [file 40425_2019_719_MOESM1_ESM.zip › Mastelic, Navarro et al_Suppl figure8.pdf]
