# Supplementary material for: Adenosine mediates functional and metabolic suppression of peripheral and tumor-infiltrating CD8+ T cells
Source: J Immunother Cancer. 2019 Oct 10;7:257. doi: 10.1186/s40425-019-0719-5 (PMC6788118; doi:10.1186/s40425-019-0719-5)
Supplement: Supplementary file 2 — Table S1. Distribution of memory subsets in total and virus-specific CD8 + T cells. Table S2: Clinical characteristics of the patients. (ZIP 61 kb) [file 40425_2019_719_MOESM2_ESM.zip › Mastelic, Navarro et al_Suppl Table1.pdf]

Table S1. Distribution of memory subsets in total and virus-specific $CD8^+$ T cells.

	T_{CM}	T_{EM}	T_{EMRA}
**Total CD8 T cells (%) - n = 12**			
**Average**	20.34	30.18	15.39
**Range**	5.86-51.6	17-52.2	4.3-53.8
**Virus-specific CD8 T cells (%) - n = 6**			
**Average**	23.87	46.45	24.47
**Range**	3.23-86.2	5.32-80.7	0-54.8
