# Supplementary material for: Adenosine mediates functional and metabolic suppression of peripheral and tumor-infiltrating CD8+ T cells
Source: J Immunother Cancer. 2019 Oct 10;7:257. doi: 10.1186/s40425-019-0719-5 (PMC6788118; doi:10.1186/s40425-019-0719-5)
Supplement: Supplementary file 2 — Table S1. Distribution of memory subsets in total and virus-specific CD8 + T cells. Table S2: Clinical characteristics of the patients. (ZIP 61 kb) [file 40425_2019_719_MOESM2_ESM.zip › Mastelic, Navarro et al_Suppl Table2.pdf]

Table S2. Clinical characteristics of the patients.

Patient	Gender	Age	Tumor type	Stage at diagnosis	Samples's origin	Other remarks
**Mel0011**	Male	40	Skin melanoma	pT3b pN0 M0, IIC	subcutaneous metastasis	BRAF mutation: previously treated with trametinib, dabrafenib, and ipilimumab
**Mel002**	Male	58	Skin melanoma	pT1a pN0 M0, IA	lymph node metastasis	
**Mel007**	Female	52	Skin melanoma	pT2 pN3 M0, IIIC	cutaneous metastasis	BRAF mutation
**Lau50**	Male	65	Melanoma of unknown primary	pT4 N2c M0, IIIB	lymph node metastasis	BRAF mutation; Isolated Limb Perfusion with Melphalan, TNF-α and IFN-γ 2 years before surgery
**Lau1015**	Male	70	Skin melanoma	pT2a N1b M1b, IV	lung metastasis	BRAF mutation; Vaccination with Melan-A natural and Tyrosinase peptides + CpG and Montanide adjuvants 1 year before surgery
**Lau1660**	Male	82	Skin melanoma	pT3b pN3 M1c, IV	lymph node metastasis	
**CRCm7**	Female	71	Colon adenocarcinoma	pT3 pN1 M1, IV	lung metastasis	KRAS mutation; Microsatellite stable
**CRCm8**	Male	68	Colon adenocarcinoma	pT4 pN2 M1, IV	liver metastasis	NRAS mutation; MSS; previously treated with Folfixiri
**CRCm9**	Male	80	Colon adenocarcinoma	pT3 pN0 M0, IIA	liver metastasis	MSS
**CRCm10**	Male	64	Colon adenocarcinoma	pT3 pN0 M1, IV	Lung metastasis	KRAS mutation; MSS; previously treated with Folfiri-bevacizumab and radiofrequency on liver metastasis
**URO334**	Male	54	no tumor	NA	Prostate	Normal prostate tissue (no BPH, no inflammation)
**URO442**	Male	70	no tumor	NA	Prostate	Chronic prostatitis + BPH
**URO476**	Male	89	prostate adenocarcinoma	Gleason 5+4=9	Prostate	Prostate cancer + BPH
**URO570**	Male	72	prostate adenocarcinoma	Gleason 3+3=6	Prostate	Prostate cancer + BPH
**URO575**	Male	83	prostate adenocarcinoma	Gleason 4+4=8	Prostate	Prostate cancer + BPH
**URO589**	Male	70	prostate adenocarcinoma	Gleason 3+3=6	Prostate	Prostate cancer + BPH
**URO372**	Male	65	no tumor	NA	Prostate	Normal prostate tissue (no BPH, no inflammation)
**URO344**	Male	73	prostate adenocarcinoma	Gleason 4+5=9	Prostate	Prostate cancer

Microsatellite stable (MSS); Benign prostate hypertrophy (BPH)
